# Supplementary material for: Dominant-negative isoform of TDP-43 is regulated by ALS-linked RNA-binding proteins
Source: J Cell Biol. 2025 Aug 8;224(10):e202406097. doi: 10.1083/jcb.202406097 (PMC12333503; doi:10.1083/jcb.202406097)
Supplement: Table S1 — is a list of PCR primer sets used in this study (for RT-PCR). [file jcb_202406097_tables1.docx]

Table S1. **List of PCR primer sets used in this study (for RT‒PCR).**

| **Gene** | **Primer Sequences** |
| --- | --- |
| *GPSM2 CE*  (Ling et al, 2015) | Forward: 5′-AGTGGACATGTGGTGGTAAGAA-3′  Reverse: 5′-GCTTCAAAGAATGACACGCCA-3′ |
| *ATG4B CE*  (Ling et al, 2015) | Forward: 5′-TGTGTCTGGATGTGAGCGTG-3′  Reverse: 5′-TCTAGGGACAGGTTCAGGACG-3′ |
| *PDP1*  (Onda-Ohto et al. 2023) | Forward: 5′-GCGTGGAAAGAGCGCCGAGC-3′  Reverse: 5′-TGCAGTGCCATAGATCCTGC-3′ |
| *BCL2L11*  (Tollervey et al. 2011) | Forward: 5′-TCTGAGTGTGACCGAGAAGG-3′  Reverse: 5′-TCTTGGGCGATCCATATCTC-3′ |
| *ACTB*  (Hasegawa-Ogawa et al, 2021) | Forward: 5′-TCACCATGGATGATGATATC-3′  Reverse: 5′-CTGGGTCATCTTCTCGCGG-3′ |
| *TARDBP*  (endogenous FL) | Forward: 5′-GTGGCTCTAATTCTGGTGCAGCAA-3′  Reverse: 5′-CACCATTCTATACCAACCAACCACAAC-3′ |
| *TARDBP*  (endogenous exon5-7) | Forward: 5′-CAGGGCCTTTGCCTTTGTTA-3′  Reverse: 5′-CAAAGACGCGGCCTGTGATG-3′ |
| *TARDBP* (FLAG)  (common in FL, MP20 and MP18) | Forward: 5′-ATGGACTACAAAGACGATGACGACAAG-3′  Reverse: 5′-CAGTCATGTCCTCTGTACA-3′ |
| *FUS* (FLAG) | Forward: 5′-ATGGACTACAAAGACGATGACGACAAG-3′  Reverse: 5′-GGAGTTGACTGAGTTCCATAG--3′ |
| *ELAVL3* (FLAG) | Forward: 5′-ATGGACTACAAAGACGATGACGACAAG-3′  Reverse: 5′-ACGTGATGATGCGGCCGTAC-3′ |
| *NOVA1* (FLAG) | Forward: 5′-ATGGACTACAAAGACGATGACGACAAG-3′  Reverse: 5′-TGATGCGATCTGGATTAACG-3′ |
| *hnRNP A1* (FLAG) | Forward: 5′-ATGGACTACAAAGACGATGACGACAAG-3′  Reverse: 5′-ACCTTGTGTGGCCTTGCATTC-3′ |
| *hnRNP K* (FLAG) | Forward: 5′-ATGGACTACAAAGACGATGACGACAAG-3′  Reverse: 5′-AATTTCTCCAATTGTTTCAATATC-3′  Reverse: 5′-CCTTTATAGTGTTGGTAATT-3′ (for deletion mutants) |
| *hnRNP E1* (FLAG) | Forward: 5′-ATGGACTACAAAGACGATGACGACAAG-3′  Reverse: 5′-GCCTGCTGGCCGCGGTACTG-3′ |
| *hnRNP E2* (FLAG) | Forward: 5′-ATGGACTACAAAGACGATGACGACAAG-3′  Reverse: 5′-GCTATTGGTCATAGAGCTGC-3′ |
| *TARDBP* mini-gene (Ex5-Int5-Ex6)  (Common in the mini-gene (Ex6)) | Forward: 5′-ATGGACTACAAAGACGATGACGACAAG-3′  Reverse: 5′-CAAAGACGCGGCCTGTGATG-3′ |
| *hnRNP A1* (endogenous) | Forward: 5′-CCCAAGCTTTCTAAGTCAGAGTCTCCTAAAG-3′  Reverse: 5′-ACCTTGTGTGGCCTTGCATTC-3′ |
| *hnRNP K* (endogenous) | Forward: 5′-TTGCGGCCGCATCCACCATGGAAACTGAACAGCCAG-3′  Reverse: 5′-AATTTCTCCAATTGTTTCAATATC-3′ |
| *TARDBP* exon 6 410-566  (Ex6 410-566) | Forward: 5′-GTGGCTCTAATTCTGGTGCAGCAA-3′  Reverse: 5′-CACCATTCTATACCAACCAACCACAAC-3′ |
