# Supplementary material for: Dominant-negative isoform of TDP-43 is regulated by ALS-linked RNA-binding proteins
Source: J Cell Biol. 2025 Aug 8;224(10):e202406097. doi: 10.1083/jcb.202406097 (PMC12333503; doi:10.1083/jcb.202406097)
Supplement: Table S2 — is a list of TaqMan Probes and primer sets used in this study (for RT-qPCR). [file jcb_202406097_tables2.docx]

Table S2. **List of TaqMan Probes and primer sets used in this study (for RT‒qPCR).**

| **Gene** | **Assay ID or Sequences of TaqMan Probe target and Primers** |
| --- | --- |
| TDP-FL  (Onda-Ohto et al., 2023) | Forward: 5′-TTTGTTCAGTGTGGAGTATATTCAGCA-3′  Reverse: 5′-AACCACTCAATATTTCAACCTTTCATG-3′  TaqMan Probe target: AAAAAGGAAGAGCTAAAGGA |
| MP20 (127) | Forward: 5′-CAGTTAGAAAGAAGTGGAAGATTTGGT-3′  Reverse: 5′-GATGCGTGATGACGAATTCTTG-3′  TaqMan Probe target: AATCCAGGAATACTGTCTACA |
| *ACTB* | Assay ID: Hs01060665_g1  (pre-designed TaqMan Gene Expression Assays (Thermo Fisher Scientific)) |
| *hnRNP K* | Assay ID: Hs03989611_gH  (pre-designed TaqMan Gene Expression Assays (Thermo Fisher Scientific)) |
| *hnRNP A1* | Assay ID: Hs01656228_s1  (pre-designed TaqMan Gene Expression Assays (Thermo Fisher Scientific)) |
| *GPSM2 CE*  (SYBR Green assay) | Forward: 5′-GGCTGATGAACAGAATGGAAG-3′  Reverse: 5′-GCTTCAAAGAATGACACGCCA-3′ |
| *ATG4B CE*  (SYBR Green assay) | Forward: 5′-TGTGTCTGGATGTGAGCGTG-3′  Reverse: 5′-CTTGCTGGCACCAATCATTG-3′ |
