# Supplementary material for: Dominant-negative isoform of TDP-43 is regulated by ALS-linked RNA-binding proteins
Source: J Cell Biol. 2025 Aug 8;224(10):e202406097. doi: 10.1083/jcb.202406097 (PMC12333503; doi:10.1083/jcb.202406097)
Supplement: Table S3 — is a list of PCR primers used in this study (for plasmid vector construction). [file jcb_202406097_tables3.docx]

Table S3. **List of PCR primers used in this study (for plasmid vector construction).**

| Constructs | **Primer Sequences** |  |
| --- | --- | --- |
| FLAG-FL  (Hasegawa-Ogawa et al, 2021) | Forward: 5′-CAAGCTTTCTGAATATATTCGGGTAAC-3′  Reverse: 5′-GGGTACCTTACATTCCCCAGCCAGAAGAC-3′ |  |
| FLAG-MP18 | Forward: 5′-CAAGCTTTCTGAATATATTCGGGTAAC-3′  Reverse: 5′-GGGTACCTTACAGCACTACTTTCAATG-3′ |  |
| FLAG-MP20 | Forward: 5′-CAAGCTTTCTGAATATATTCGGGTAAC-3′  Reverse: 5′-GGGTACCTCAAAGACGCGGCCTGTGATG-3′ |  |
| FLAG-FUS | Forward: 5′-CCCAAGCTTGCCTCAAACGATTATACC-3′  Reverse: 5′-CGGGGTACCTTAATACGGCCTCTCCCTG-3′ |  |
| FLAG-ELAVL3 | Forward: 5′-CAAGCTTGTCACTCAGATACTGG-3′  Reverse: 5′-GGGTACCTCACGCCTTGTGCTGTTTGC-3′ |  |
| FLAG-NOVA1 | Forward: 5′-CCCAAGCTTATGGCGGCAGCTCCCATC-3′  Reverse: 5′-CGGGGTACCTCAACCCACTTTCTGAGGATTG-3′ |  |
| FLAG-hnRNP A1 | Forward: 5′-CCCAAGCTTTCTAAGTCAGAGTCTCCTAAAG-3′  Reverse: 5′-CGGGGTACCTTAAAATCTTCTGCCACTG-3′ |  |
| FLAG-hnRNP E1 | Forward: 5′-CCCAAGCTTGATGCCGGTGTGACTGAAAG-3′  Reverse: 5′-GGGGTACCCTAGCTGCACCCCATGCCCTTC-3′ |  |
| TDP-FL-Venus  (Hasegawa-Ogawa et al. 2021) | Forward1: 5′-TGGGCCCCCACCATGTCTGAATATATTCGGGTAAC-3′  Reverse1: 5′-GGGTACCCATTCCCCAGCCAGAAGAC-3′  Forward2: 5′-CGGATCCGTGAGCAAGGGCGAGG-3′  Reverse2: 5′-CAAGCTTTTACTTGTACAGCTCGTCCATG-3′ |  |
| MP18-Venus | Forward1: 5′-GGGTACCGAAAAGTAAAAGATGTCTG-3′  Reverse1: 5′-CGGATCCCAGCACTACTTTCAATGAAG-3′  Forward2: 5′-CGGATCCGTGAGCAAGGGCGAGG-3′  Reverse2: 5′-GGAATTCTTACTTGTACAGCTCGTCC-3′ |  |
| MP20-Venus | Forward1: 5′-GGGTACCGAAAAGTAAAAGATGTCTG-3′  Reverse1: 5′-CGGATCCAAGACGCGGCCTGTGATG-3′  Forward2: 5′-CGGATCCGTGAGCAAGGGCGAGG-3′  Reverse2: 5′-GGAATTCTTACTTGTACAGCTCGTCC-3′ |  |
| FLAG-hnRNP E2 | Forward: 5′-CCCAAGCTTGACACCGGTGTGATTGAAG-3′  Reverse: 5′-GGGGTACCCTAGCTGCTCCCCATG-3′ |  |
| *TARDBP* mini-gene  (Ex5-Int5-Ex6) | Forward1: 5′-CCCAAGCTTGCCACCATGGACTACAAAGACGATG-3′  Reverse1: 5′-GGGGTACCCTTGTCGTCATCGTCTTTGTA-3′  Forward2: 5′-GGGGTACCCAAAGCCAAGATGAGCC-3′  Reverse2: 5′-CGGGATCCTCCCCCTGGAGGCTG-3′  Forward3: 5′-CGGGATCCTCCAGCTTCAGCCTC-3′  Reverse3: 5′-CACGATATCAATTGTAGTGTGTTGGTTTACCAATCAGGC-3′ |  |
| *TARDBP* mini-gene  (Ex6) | Forward1: 5′-GGGGTACCGCCACCATGGACTACAAAGACGATG-3′  Reverse1: 5′-CCCAAGCTTGTCGTCATCGTCTTTGT-3′  Forward2: 5′-CCCAAGCTTATTGCGCAGTCTCTTTGTG-3′  Reverse2: 5′-CTCGAGGTCAATGCATGACAGGTGATG-3′ |  |
| Deletion of hnRNP K potential consensus (Ex6 262-266) | Forward: 5′-ATGATGGCTGCCGGCAGCACTACAGAGCAGT-3′  Reverse: 5′-GTAGTGCTGCCGGCAGCCATCATGGCTGGAT-3′ |  |
| Deletion of hnRNP K potential consensus (Ex6 329-333) | Forward: 5′-CAGTCAGTCGGGTAATAACCAAAACCAAGGCAA-3′  Reverse: 5′-TTACCCGACTGACTGGTTCTGCTGGCTGGCTAA-3′ |  |
| Deletion of hnRNP A1 potential consensus (Int5 414-419) | Forward: 5′-GTAAGACGGTATGGGCCTAGATGTTTGTGATAC-3′  Reverse: 5′-CCCATACCGTCTTACAAGGTCAGAAAACTTAGC-3′ |  |
| KH1 deletion mutant of  FLAG-hnRNP K (Del KH1) | Forward: 5′-AGAAATTCTGAAGAAAATCATCCCTACCTTGGAAG-3′  Reverse: 5′-TTCTTCAGAATTTCTAATTCAACCATCTCATCAG-3′ |  |
| KH2 deletion mutant of  FLAG-hnRNP K (Del KH2) | Forward: 5′-GTTGAGGAAACCCGATAGGGTTGTAGAGTGCAT-3′  Reverse: 5′-TCGGGTTTCCTCAACTCGCAGTCAAAGTCAC-3′ |  |
| KH3 deletion mutant of  FLAG-hnRNP K (Del KH3) | Forward: 5′-ATTACTACAGCAGATGTTGAAGGATTCTAA-3′  Reverse: 5′-ATCTGCTGTAGTAATAATAGGTCCACCAAG-3′ |  |
| KI and KNS deletion mutant of  FLAG-hnRNP K (Del KI/KNS) | Forward: 5′-TGGTTTTGAACCACAGGGTGGCTCC-3′  Reverse: 5′-TGTGGTTCAAAACCACCATAATCATAGGTTTCATC-3′ |  |
| FLAG-hnRNP K  with KH1 mutations (KH1mt) | Forward: 5′-TTGGAGACGACGGCAAGAATATTAAGGCT-3′  Reverse: 5′-TTGCCGTCGTCTCCAATCACTGCCCCAGC-3′ |  |
| FLAG-hnRNP K  with KH2 mutations (KH2mt) | Forward: 5′-TTGGGGACGACGGTGCTAAAATCAAAGAA-3′  Reverse: 5′-GCACCGTCGTCCCCAATAATTCCTCCTGC-3′ |  |
| FLAG-hnRNP K  with KH3 mutations (KH3mt) | Forward: 5′-TTGGCGACGACGGTCAGCGGATTAAAC-3′  Reverse: 5′-GACCGTCGTCGCCAATAATAGATCCAGCCAAATC-3′ |  |
| FLAG-TDP-FL, MP20, MP18  with RRM1 mutations (Rmt)  (Hasegawa-Ogawa et al. 2021) | Forward: 5′-AGGGGCTGGGCCTGGTTCGTTTTACGGAATATG-3′  Reverse: 5′-GAACCAGGCCCAGCCCCTTTGAATGACCAGTCT-3′ |  |
| FLAG-hnRNP A1  with RRM1 mutations (R1mt) | Forward: 5′-GGCGACGGGGACGTCACATATGCCACTGTGGAG-3′  Reverse: 5′-GACGTCCCCGTCGCCCCTGGAGCGCTTGGTG-3′ |  |
| FLAG-hnRNP A1  with RRM2 mutations (R2mt) | Forward: 5′-GGCGACGCCGACGTAACCTTTGACGACCATG-3′  Reverse: 5′-TACGTCGGCGTCGCCCCTTTTCTTGCCAC-3′ |  |
| FLAG-FUS (P525L) | Forward: 5′-GAGAGGCTGTATTAAGGTACCAGTCGAC-3′  Reverse: 5′-TTAATACAGCCTCTCCCTGCGATCC-3′ |  |
| Venus-3'UTR | Forward1: 5′-CGGGGTACCCCACCATGGTGAGCAAGGGCGAG-3′  Reverse1: 5′-CGCGGATCCTTACTTGTACAGCTCGTC-3′  Forward2: 5′-GCGGATCCCGGTGGGTGTCCCATTTTTATCCG-3′  Reverse2: 5′-CCGCTCGAGCTGCAAAACAAAGACAC-3′ |  |
| Venus | Forward: 5′-CGGGGTACCCCACCATGGTGAGCAAGGGCGAG-3′  Reverse: 5′-CCTCGAGTTACTTGTACAGCTCGTC-3′ |  |
| *TARDBP* mini-gene  (Ex5-Int5-Ex6), (Ex6)  Del 185-284 | Forward: 5′-GTTTGGTTGGGGTATGATGGGCATG-3′  Reverse: 5′-CATACCCCAACCAAACCAGCTCCAC-3′ |  |
| *TARDBP* mini-gene  (Ex5-Int5-Ex6), (Ex6)  Del 285-384 | | Forward: 5′-CAGAGCAGTTCGGTTCTGGAAATAACTC-3′  Reverse: 5′-GAACCGAACTGCTCTGTAGTGCTGC-3′ |
| *TARDBP* mini-gene  (Ex5-Int5-Ex6), (Ex6)  Del 385-484 | | Forward: 5′-CAGGCCGCTTTGGCTCAAGCATGG-3′  Reverse: 5′-CCAAAGCGGCCTGGTTTGGCTCC-3′ |

| *TARDBP* mini-gene  (Ex5-Int5-Ex6), (Ex6)  Del 485-584 | Forward: 5′-AATGGAGAACTCATGGTAAGTATATTG-3′  Reverse: 5′-CATGAGTTCTCCATTAAAACCACTG-3′ |
| --- | --- |
| *TARDBP* mini-gene  (Ex5-Int5-Ex6), (Ex6)  Del 585-662 | Forward: 5′-TTCTAAGCAGTATTTTTGACAT-3′  Reverse: 5′-ATACTGCTTAGAAAAATTTGAATTC-3′ |
| *TARDBP* mini-gene (Ex6)  Del 663-762 | Forward: 5′-ATATTCACTGCTGTTTGCCTGATTG-3′  Reverse: 5′-AGCAGTGAATATACTCCACACTGAAC-3′ |
| *TARDBP* mini-gene  (Ex5-Int5-Ex6), (Ex6)  Del 763-865 | Forward: 5′-AAAGTGATTCAAAACGGAAACCATTG-3′  Reverse: 5′-CGTTTTGAATCACTTTCAACCACTC-3′ |
| *TARDBP* mini-gene  (Ex5-Int5-Ex6), (Ex6)  Del 866-985 | Forward: 5′-CTTTGCATGTTTGGTTCTTTTGTTTTG-3′  Reverse: 5′-GAACCAAACATGCAAAGAATTCACTTG-3′ |
| *TARDBP* mini-gene (Ex6)  Del 485-531 | Forward: 5′-TAATGGAGACAGTGGGGTTGTGGTTGGTTG-3′  Reverse: 5′-CCACTGTCTCCATTAAAACCACTGCCCGAC-3′ |
| *TARDBP* mini-gene (Ex6)  Del 532-584 | Forward: 5′-GCTGGGGAATGTAGAACTCATGGTAAGTATATTG-3′  Reverse: 5′-ATGAGTTCTACATTCCCCAGCCAGAAGAC-3′ |
| *TARDBP* mini-gene (Ex6)  Del 535-555 | Forward: 5′-GGAATGTAGACAATAGAATGGTGGGAATTC-3′  Reverse: 5′-ATTCTATTGTCTACATTCCCCAGCCAG-3′ |
| *TARDBP* mini-gene (Ex6)  Del 556-568 | Forward: 5′-TGGTTGGTAATTCAAATTTTTCTAAACTC-3′  Reverse: 5′-TTGAATTACCAACCAACCACAAC-3′ |

| *TARDBP* mini-gene  (Ex5-Int5-Ex6)  Del int5 51-250 | Forward: 5′-CTCATGCTGTGGCAGTGAACTGAGATCACAC-3′  Reverse: 5′-ACTGCCACAGCATGAGCCACCAC-3′ |
| --- | --- |
| *TARDBP* mini-gene  (Ex5-Int5-Ex6)  Del int5 251-450 | Forward: 5′-GAGGCGGAGCTGATATCACAAATACAAC-3′  Reverse: 5′-TGATATCAGCTCCGCCTCCTGGGTTC-3′ |
| *TARDBP* mini-gene  (Ex5-Int5-Ex6)  Del int5 451-650 | Forward: 5′-ACCTGTATTGGCTCAGCCTCCTGAG-3′  Reverse: 5′-AGGCTGAGCCAATACAGGTATCACAAACATC-3′ |
| *TARDBP* mini-gene  (Ex5-Int5-Ex6)  Del int5 651-828 | Forward: 5′-GATTGTCTACCACGCCTGGCCAG-3′  Reverse: 5′-CGTGGTAGACAATCGCTTGAACCCATG-3′ |
| *TARDBP* mini-gene  (Ex5-Int5-Ex6)  Del int5 791-965 | Forward: 5′-CTGCCCATACTGCTCCTGCTCCCGGC-3′  Reverse: 5′-GAGCAGTATGGGCAGATCACCTGAG-3′ |
| *TARDBP* mini-gene  (Ex5-Int5-Ex6)  Del int5 966-1054 | Forward: 5′-GTGAGCCATCATAGCTCACTACACC-3′  Reverse: 5′-GCTATGATGGCTCACGCCTGTAATC-3′ |
| *TARDBP* mini-gene  (Ex5-Int5-Ex6)  Del int5 1055-1274 | Forward: 5′-TGGTGTACCCAAAGCGCTAGGATTAC-3′  Reverse: 5′-GCTTTGGGTACACCACTGTGTTCCAC-3′ |
| *TARDBP* mini-gene  (Ex5-Int5-Ex6)  Del int5 1275-1474 | Forward: 5′-CTTGGCCTTATGAATCAGTGGTTTAATC-3′  Reverse: 5′-GATTCATAAGGCCAAGGTGGGAG-3′ |
