# Supplementary figures and images for: Dominant-negative isoform of TDP-43 is regulated by ALS-linked RNA-binding proteins
Source: J Cell Biol. 2025 Aug 8;224(10):e202406097. doi: 10.1083/jcb.202406097 (PMC12333503; doi:10.1083/jcb.202406097)

# Source Data F1

**B**

TDP-43

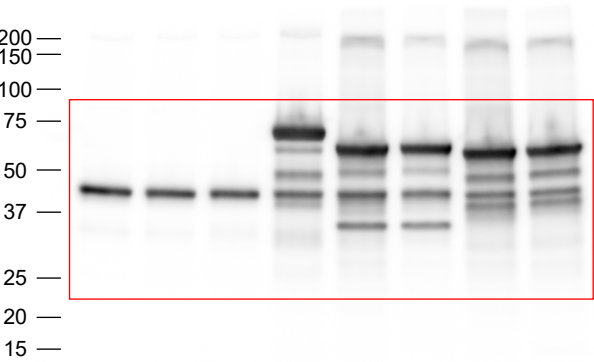

Venus

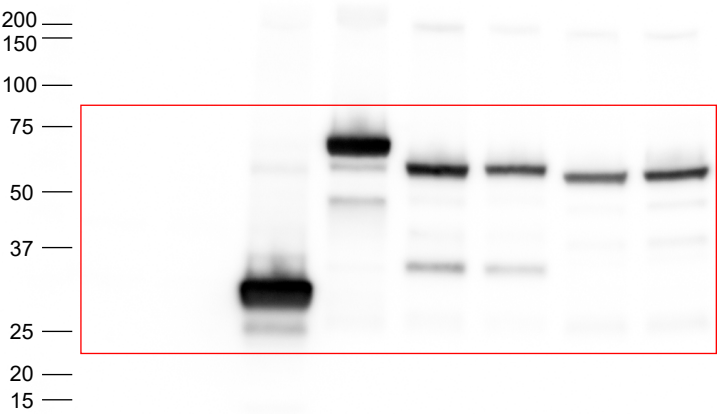

$\beta$ -Actin (reprobed following Venus detection)

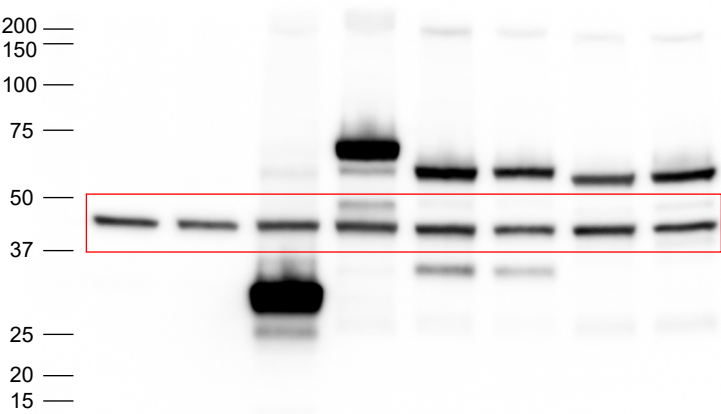

Supplement: SourceData F1 — is the source file for Fig. 1. [file jcb_202406097_sourcedataf1.pdf]

Source Data F3

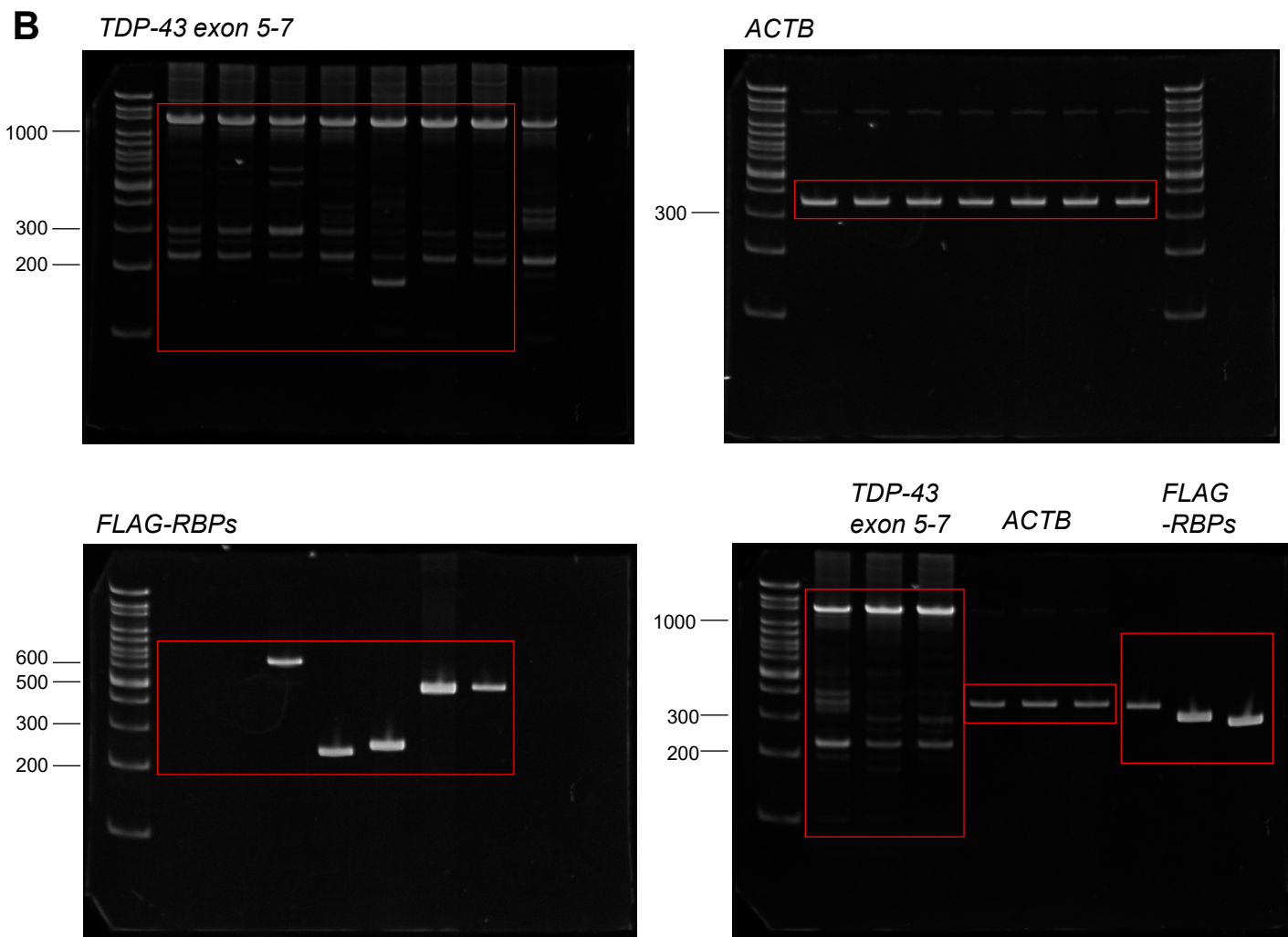

Source Data F3

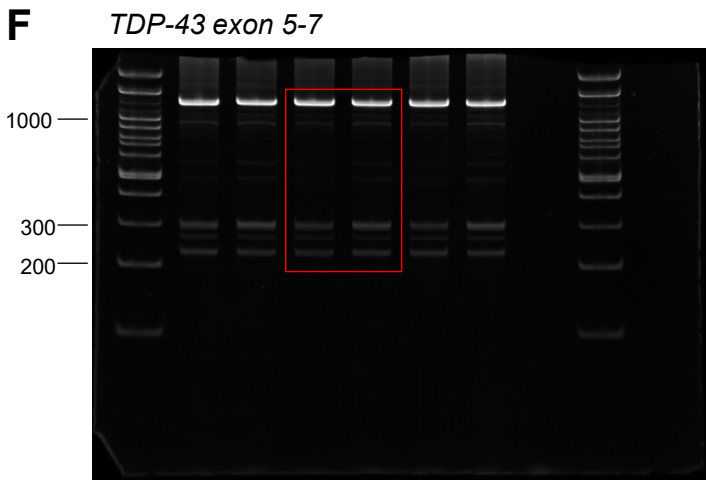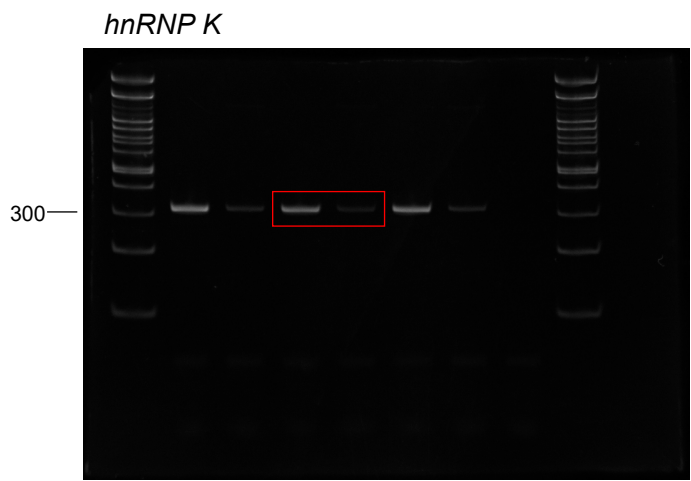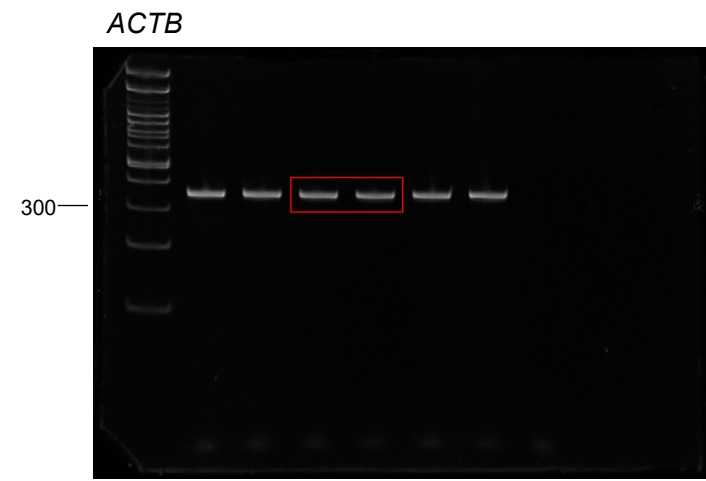

Supplement: SourceData F3 — is the source file for Fig. 3. [file jcb_202406097_sourcedataf3.pdf]

Source Data F4

D TDP-43

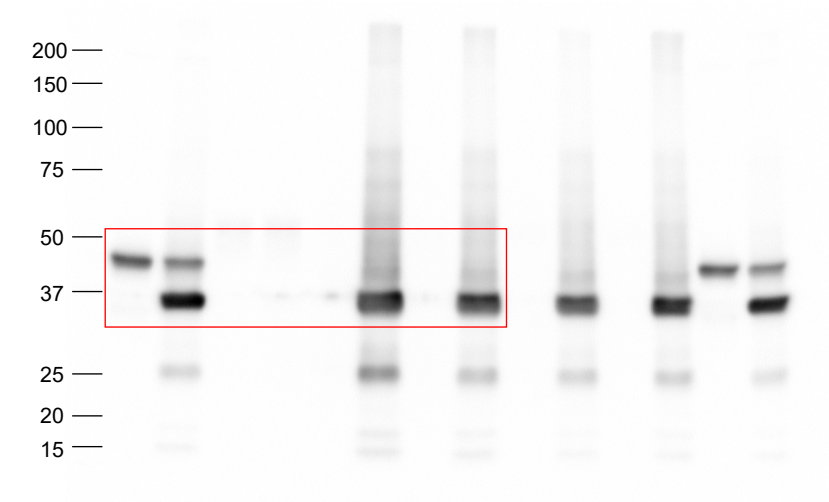

MP20

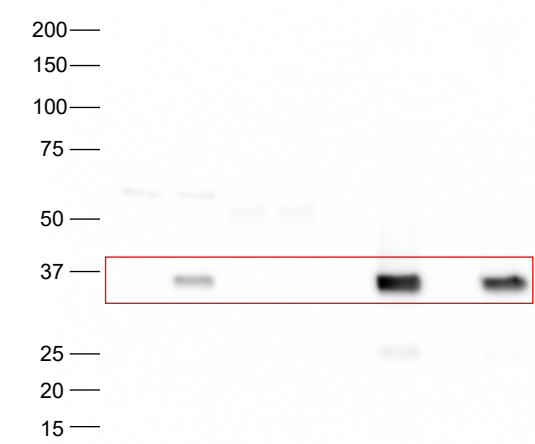

$\beta$ -Actin  
(reprobed following MP20 detection)

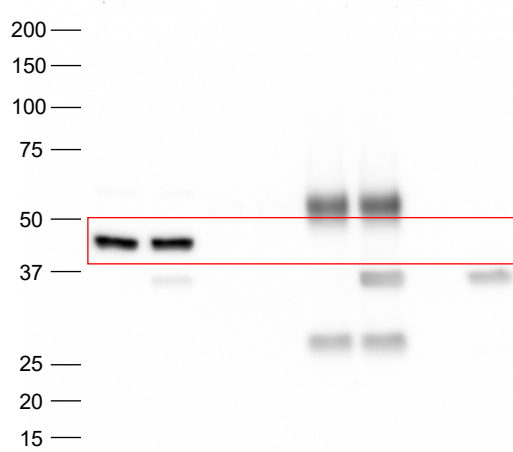

FLAG

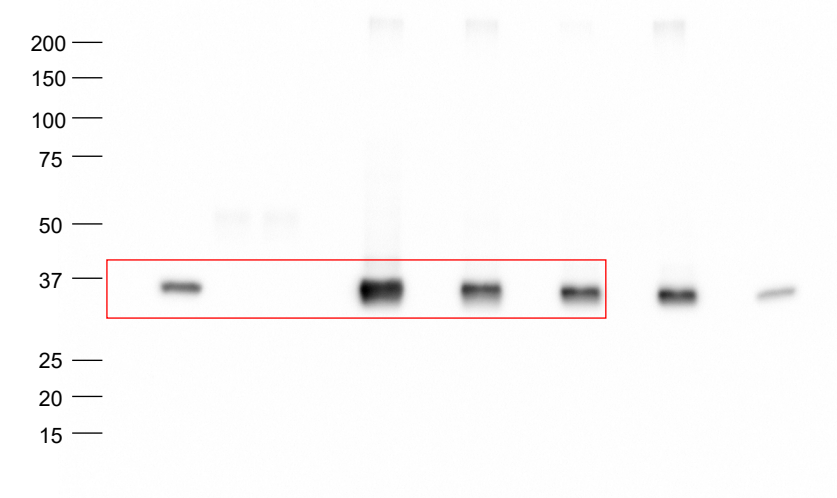

# Source Data F4

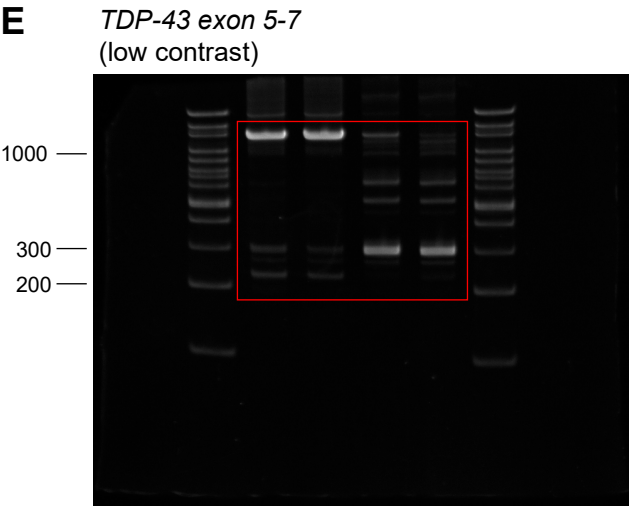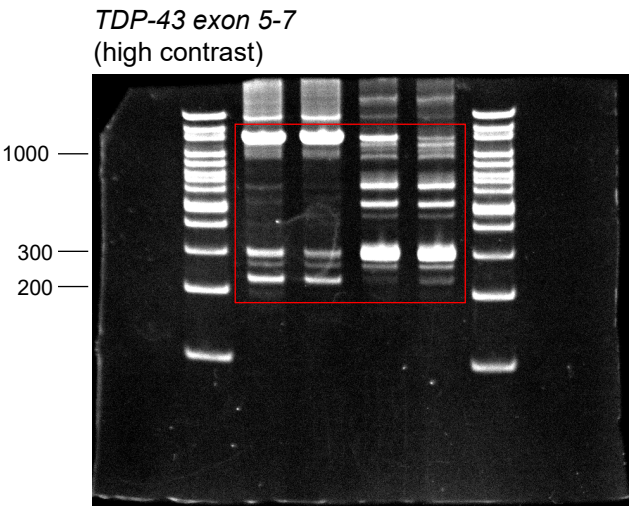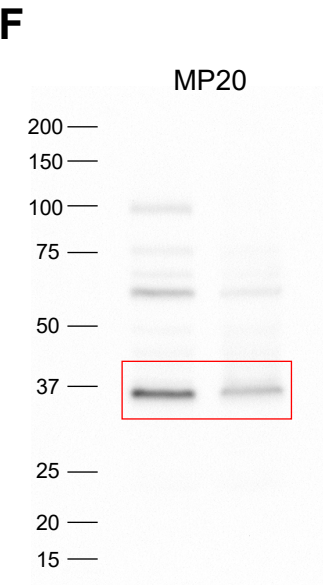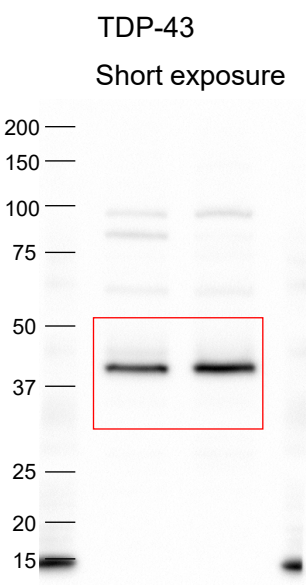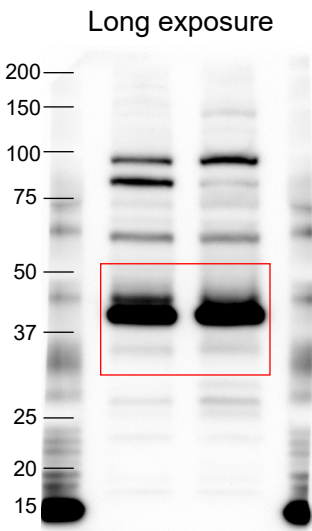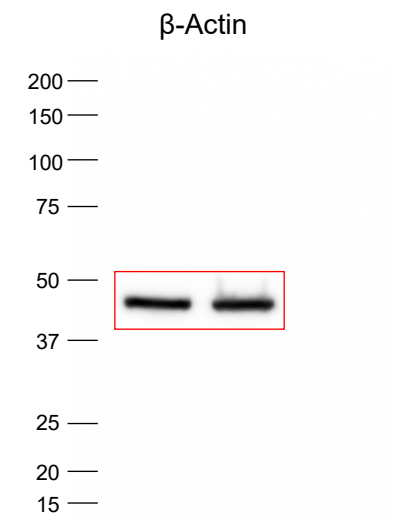

Supplement: SourceData F4 — is the source file for Fig. 4. [file jcb_202406097_sourcedataf4.pdf]

Source Data FS3

**B**

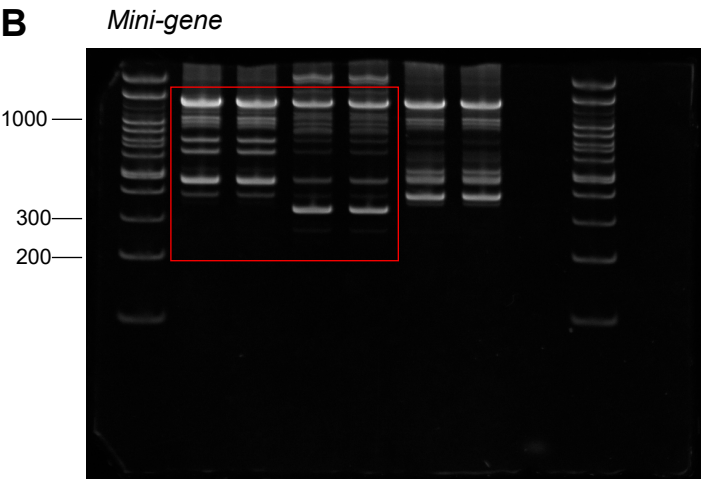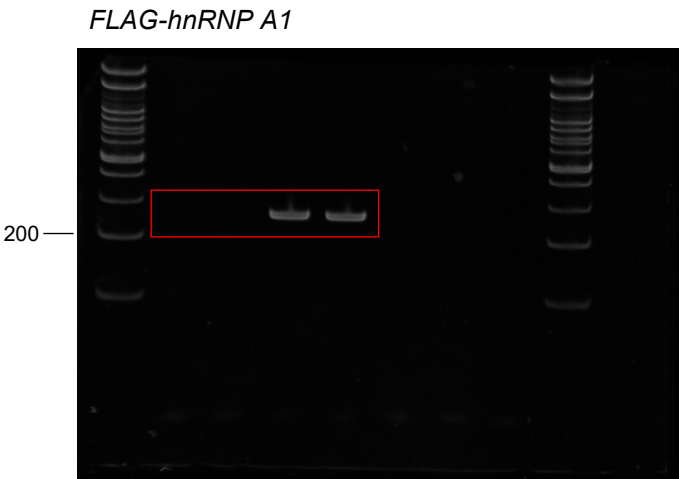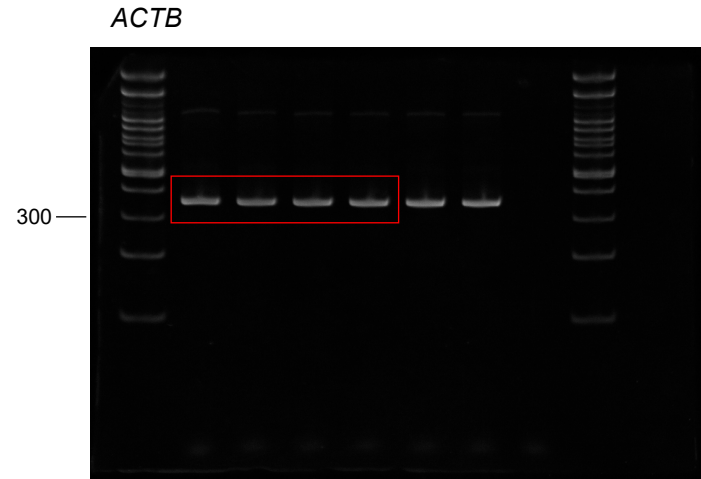

**D**

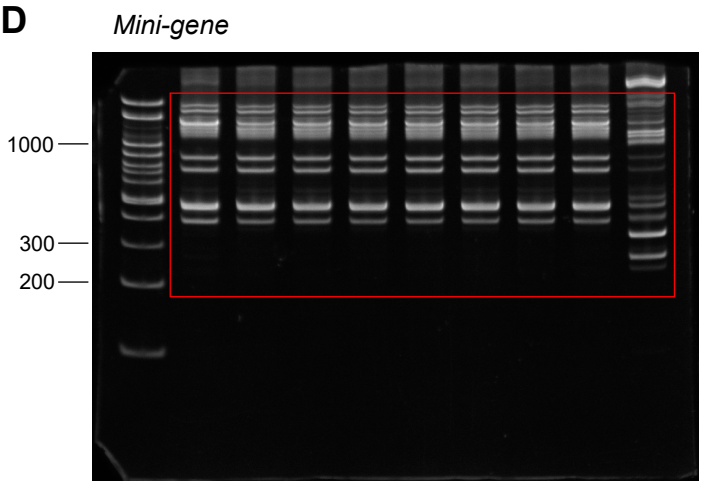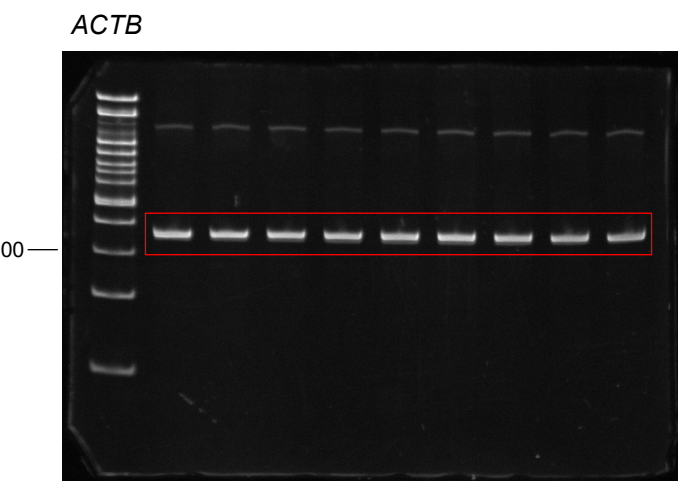

Source Data FS3

D

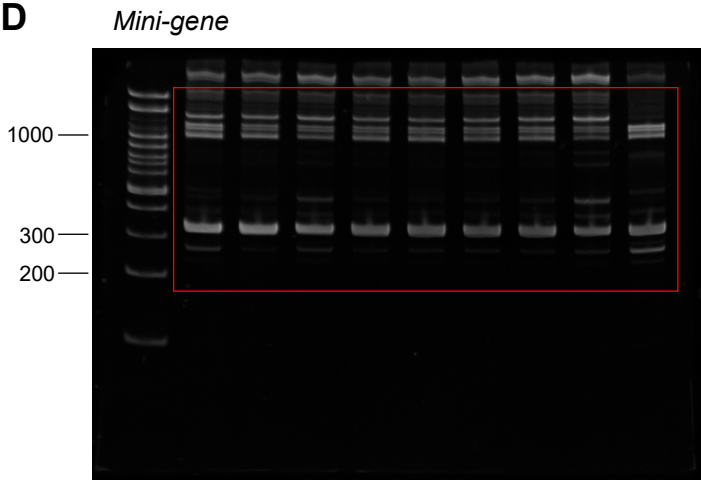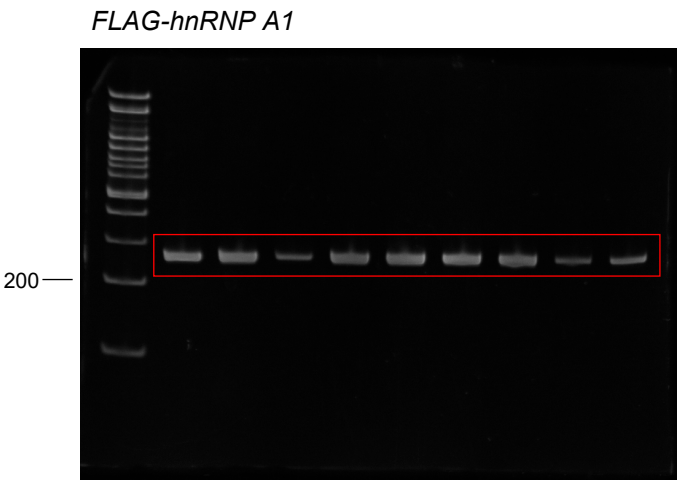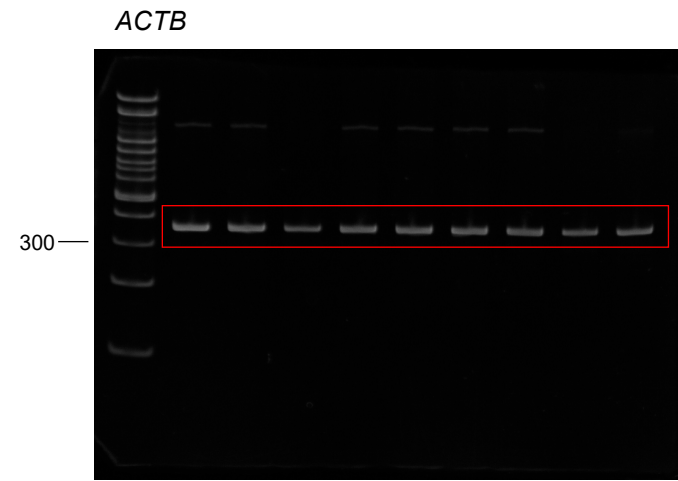

F

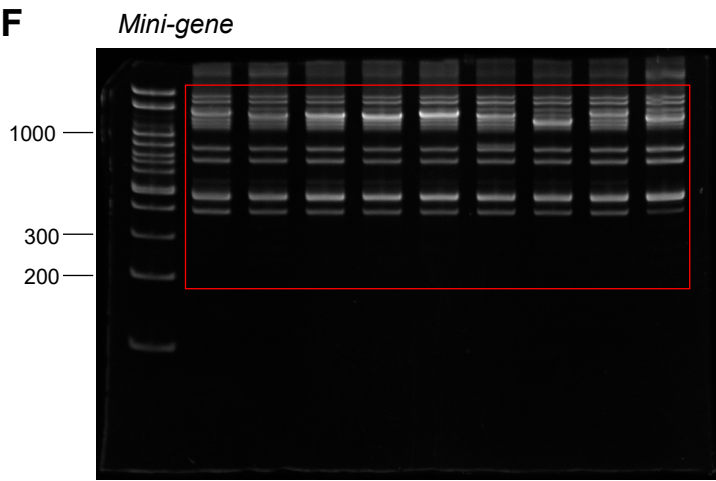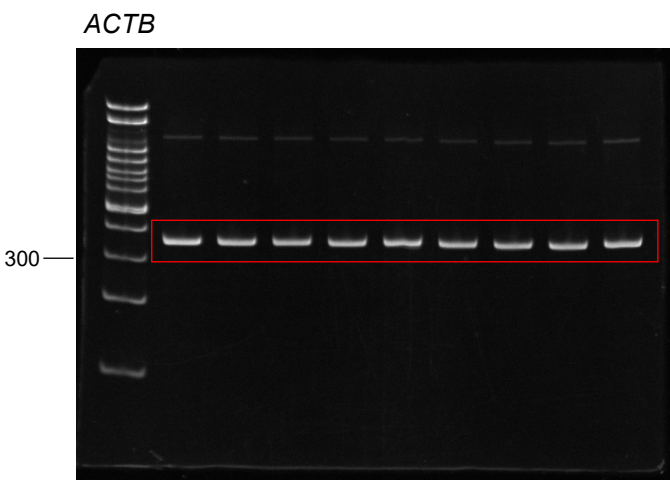

Source Data FS3

F

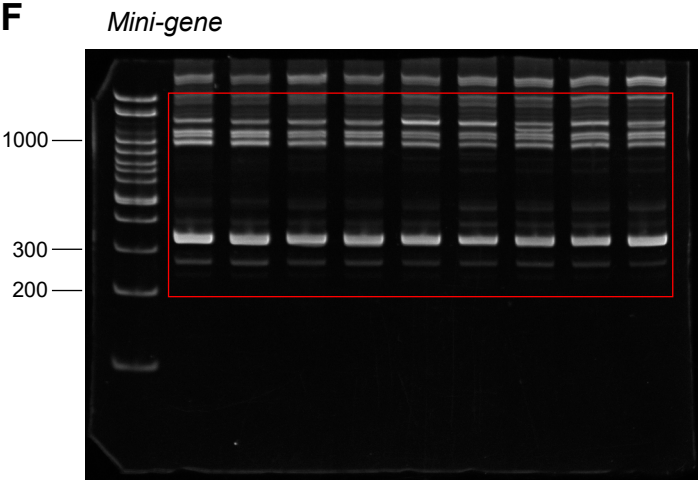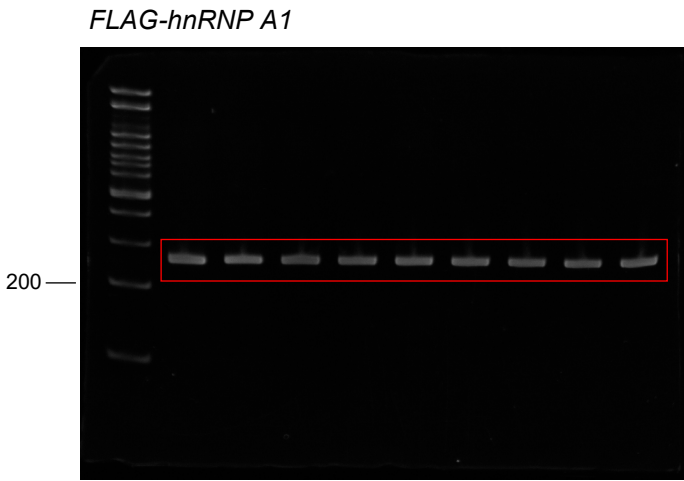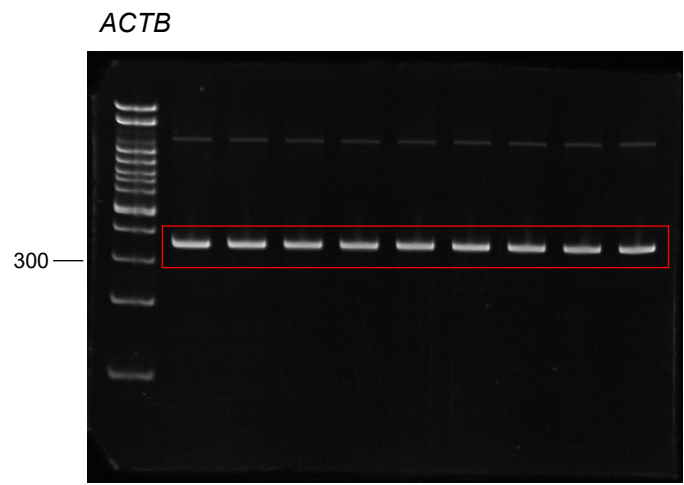

Supplement: SourceData FS3 — is the source file for Fig. S3. [file jcb_202406097_sourcedatafs3.pdf]

Source Data FS4

**B**

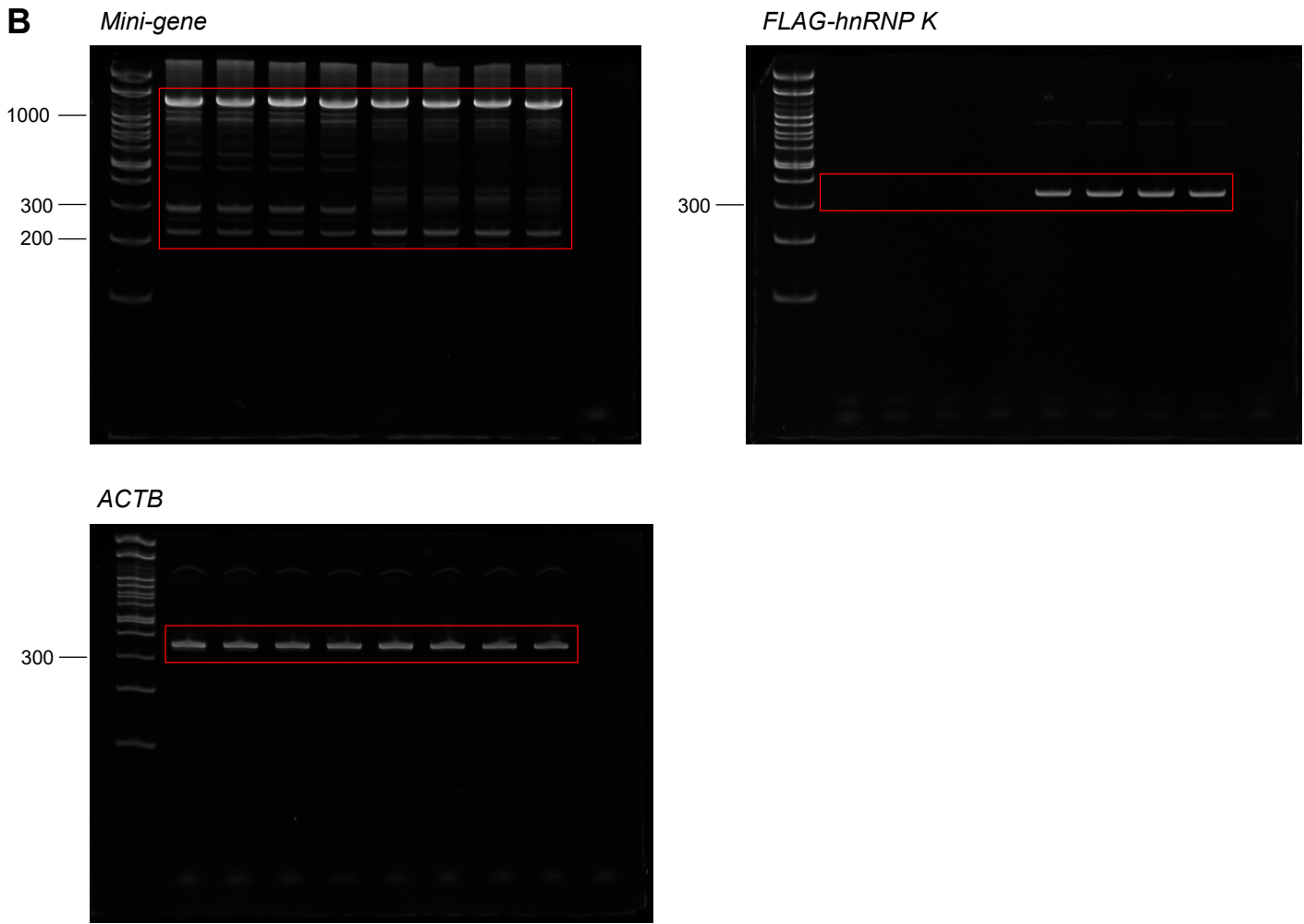

**D**

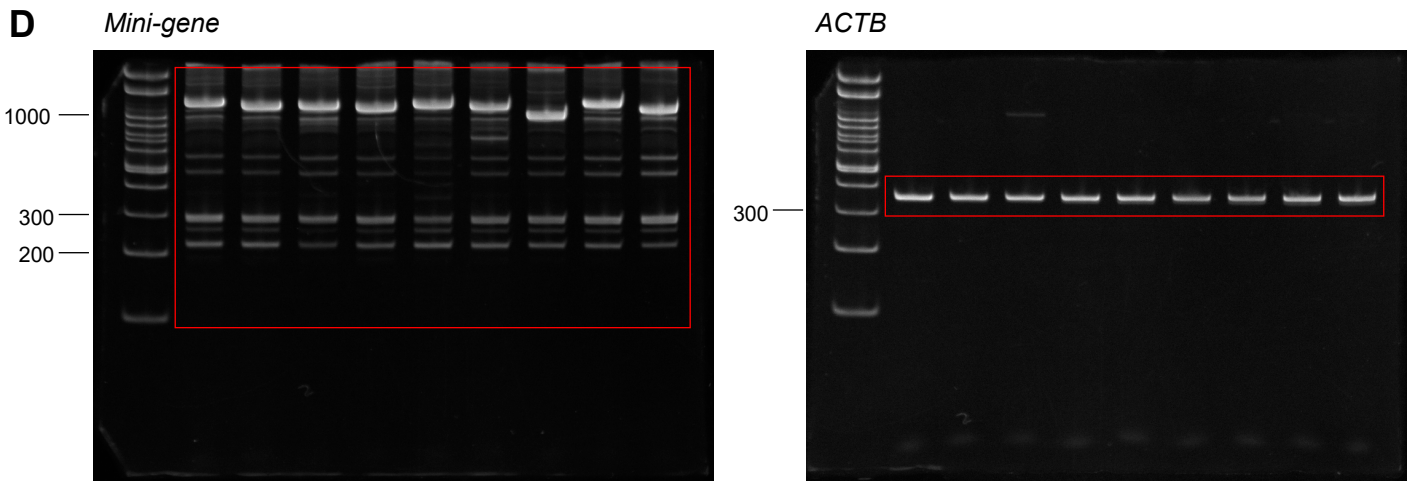

Source Data FS4

**D**     *Mini-gene*

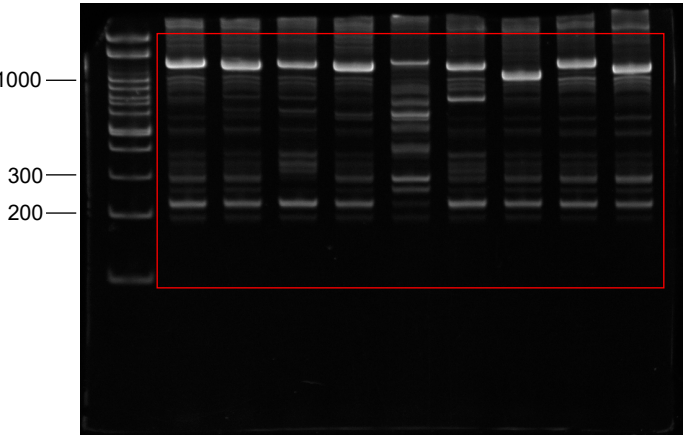

*FLAG-hnRNP K*

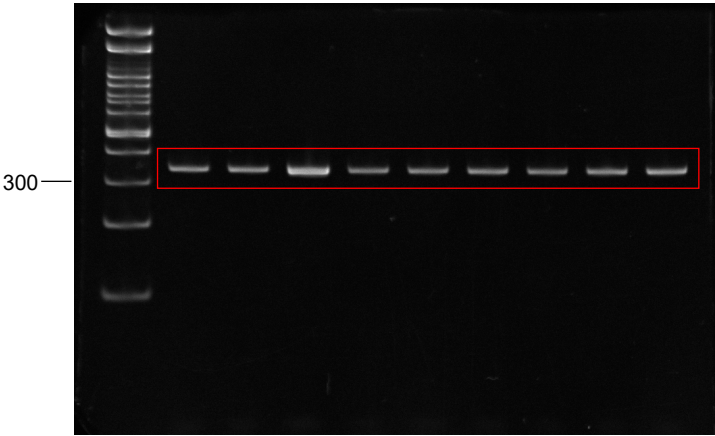

*ACTB*

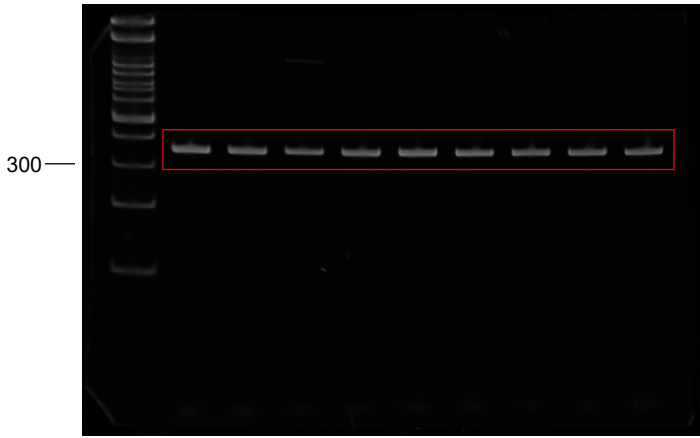

Source Data FS4

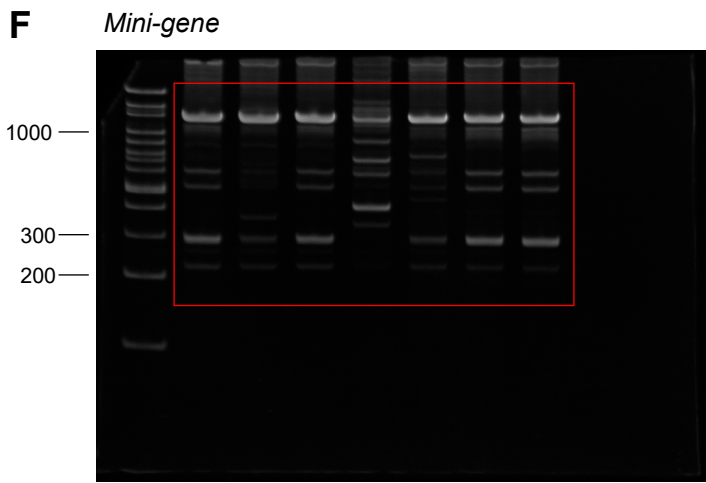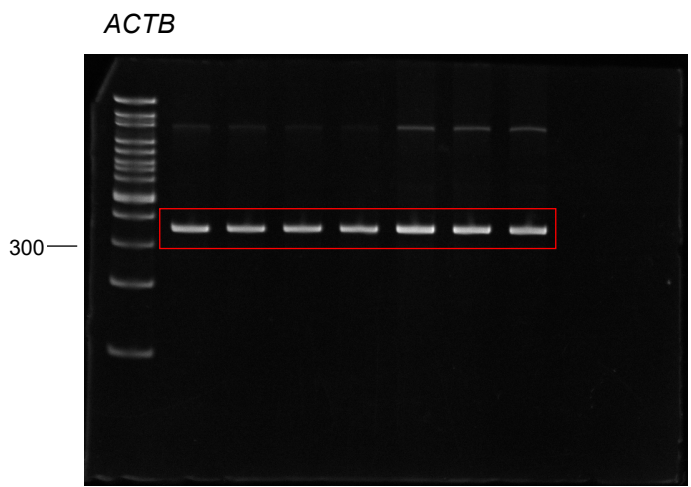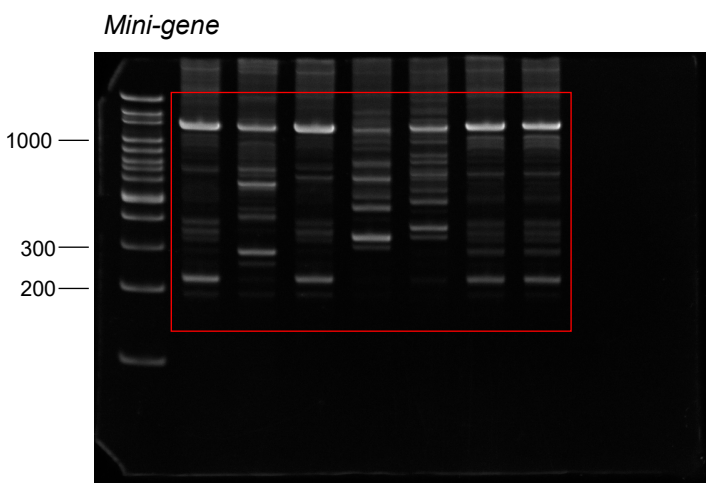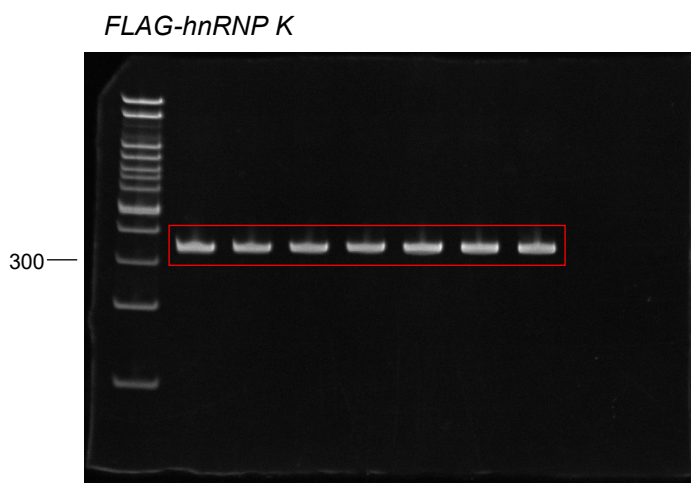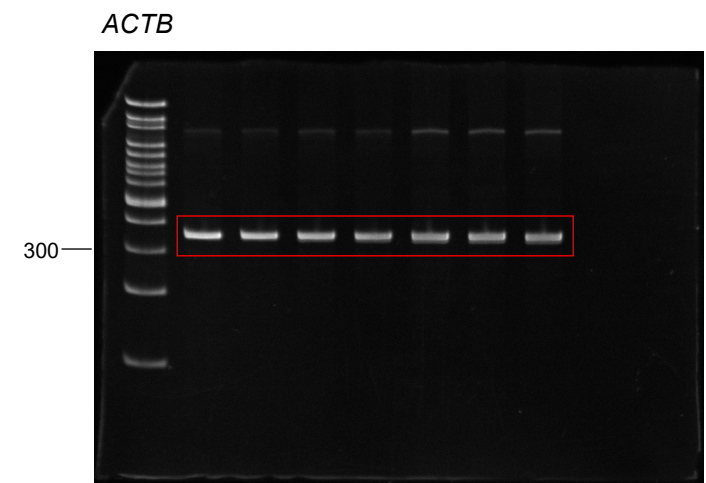

Supplement: SourceData FS4 — is the source file for Fig. S4. [file jcb_202406097_sourcedatafs4.pdf]
