# Supplementary material for: Dominant-negative isoform of TDP-43 is regulated by ALS-linked RNA-binding proteins
Source: J Cell Biol. 2025 Aug 8;224(10):e202406097. doi: 10.1083/jcb.202406097 (PMC12333503; doi:10.1083/jcb.202406097)

Source Data F2

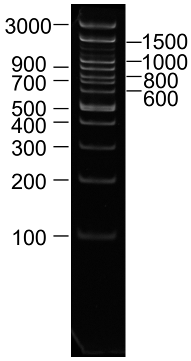

A *GPSM2*

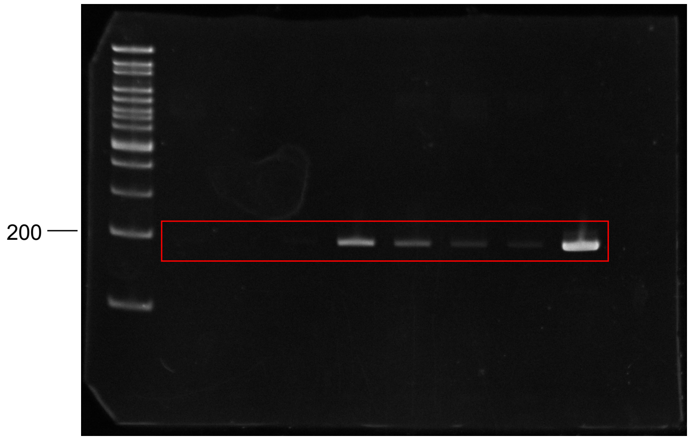

ATG4B

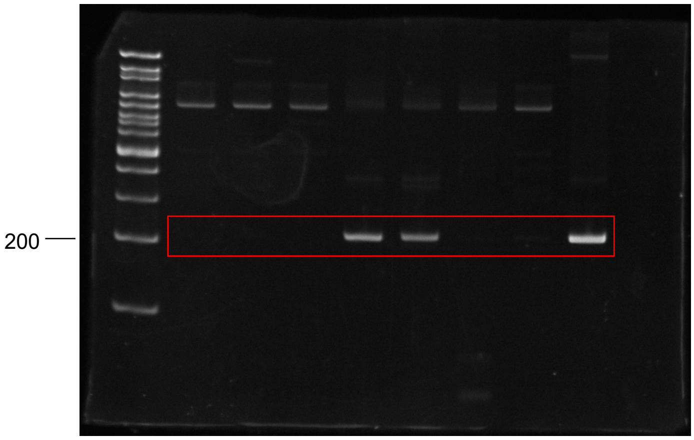

PDP1

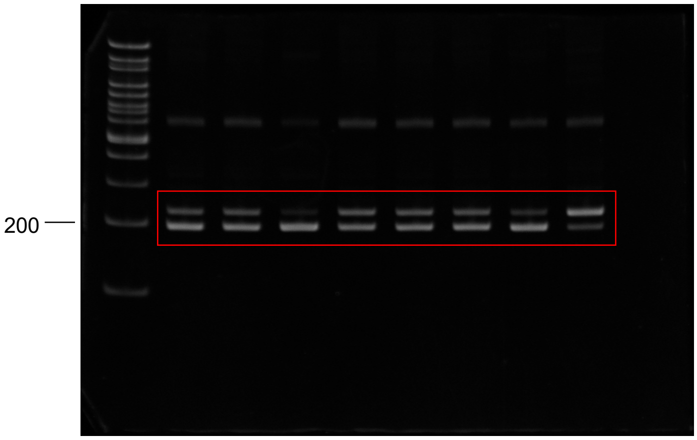

BCL2L11

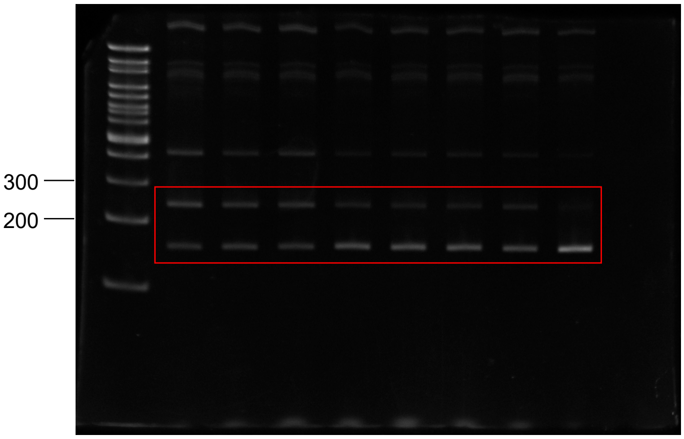

FLAG

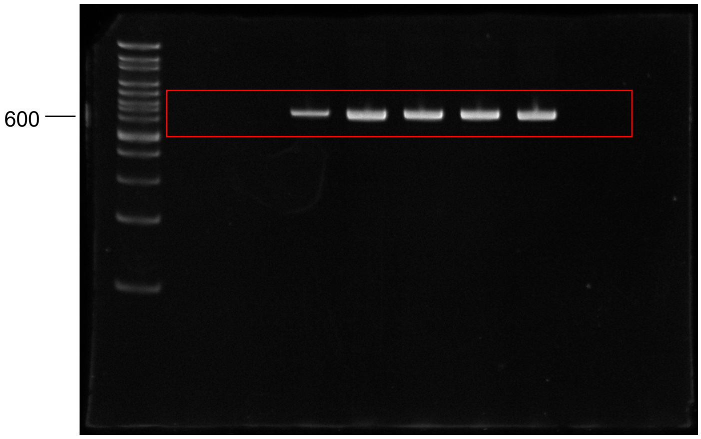

Source Data F2

A

FL-endo

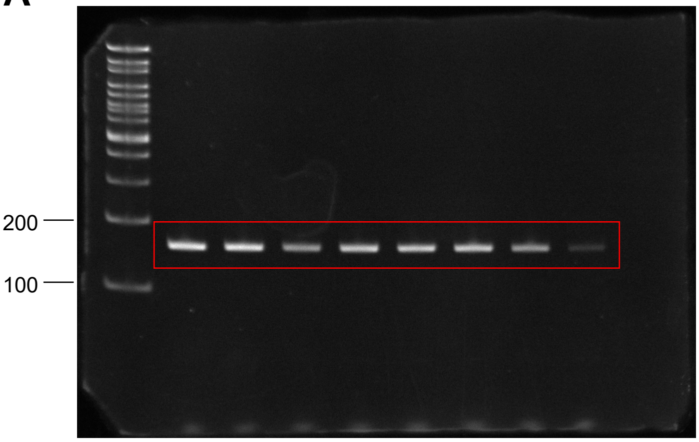

ACTB

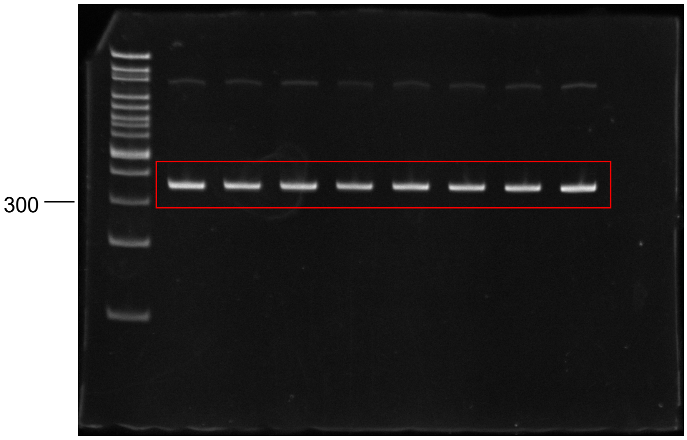

E

GPSM2

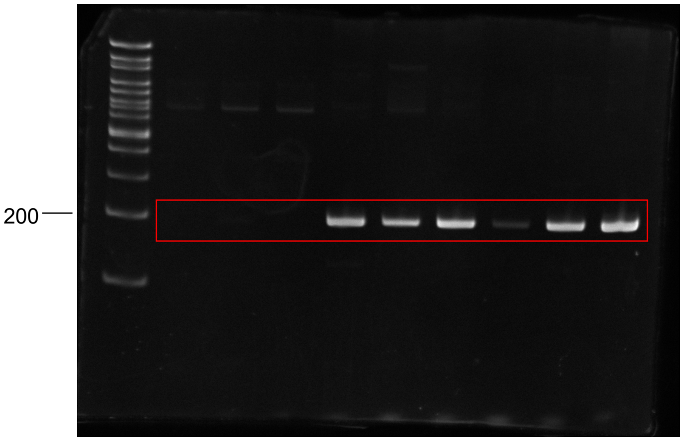

ATG4B

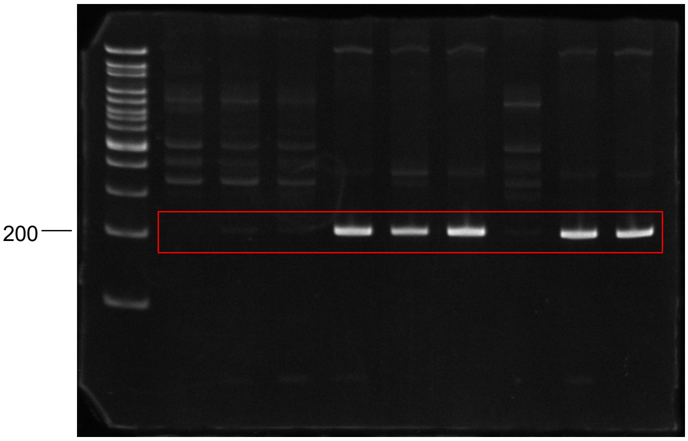

PDP1

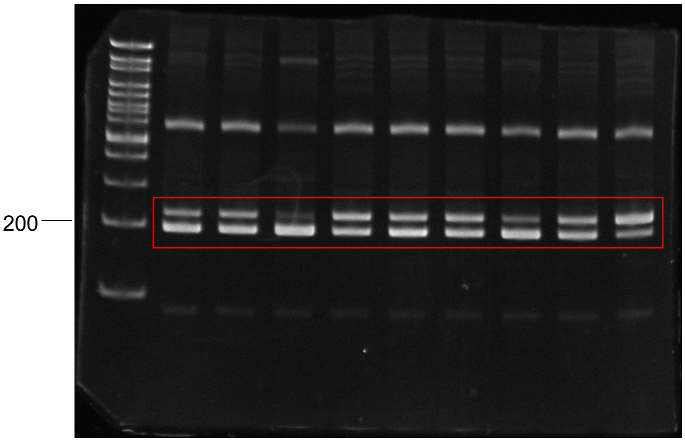

BCL2L11

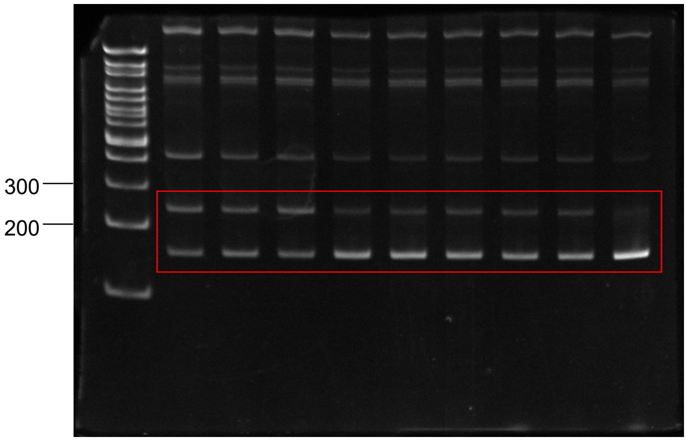

Source Data F2

E

FLAG

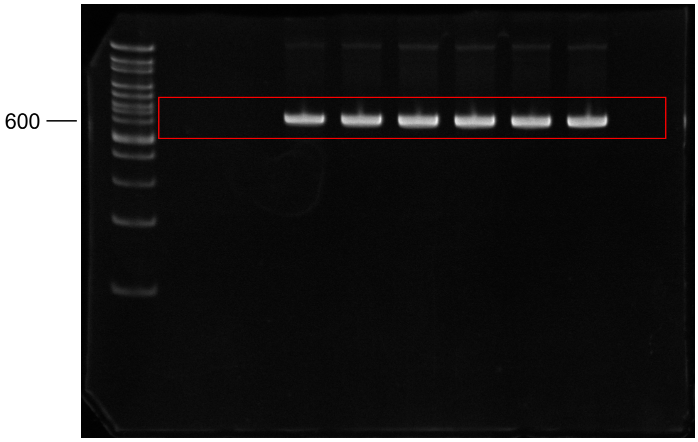

FL-endo

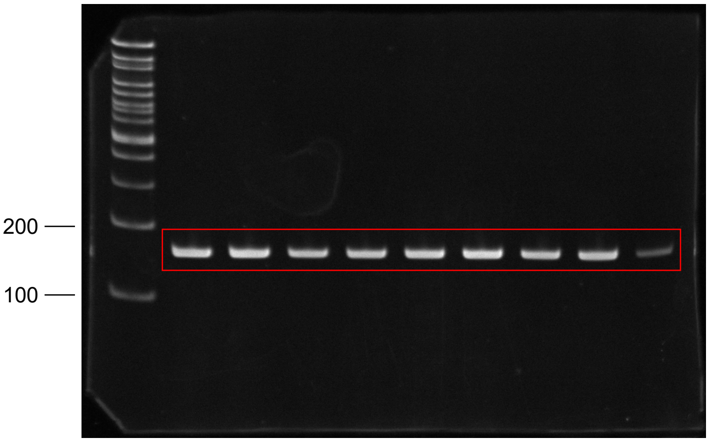

ACTB

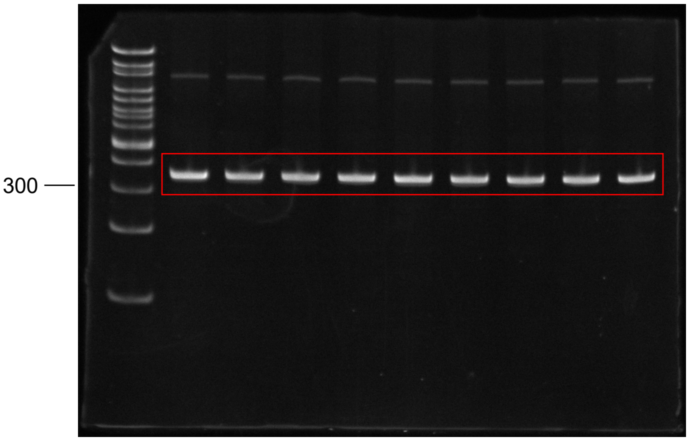

Source Data F2

H IP: FLAG

Venus

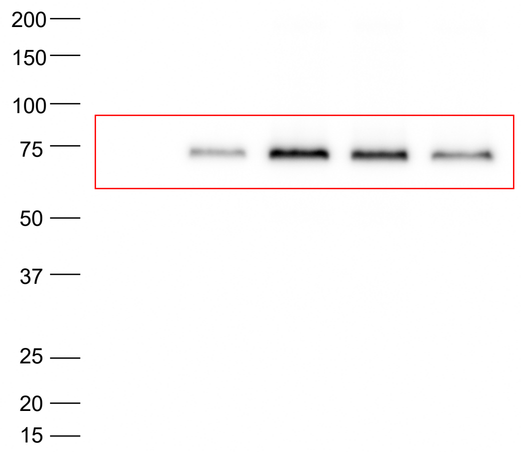

FLAG

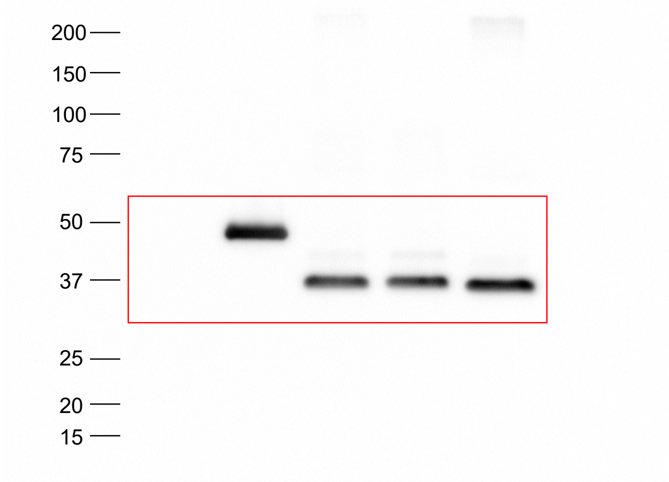

Input

Venus

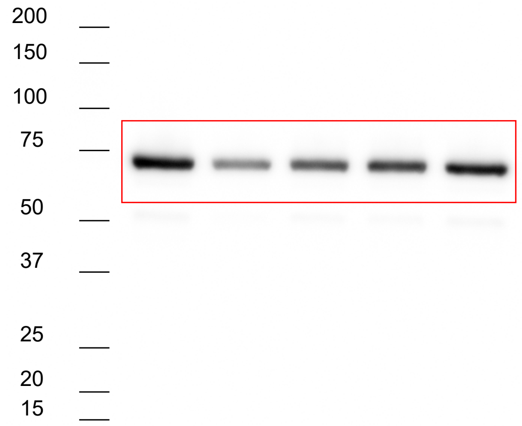

β-Actin
(reprobed following Venus detection)

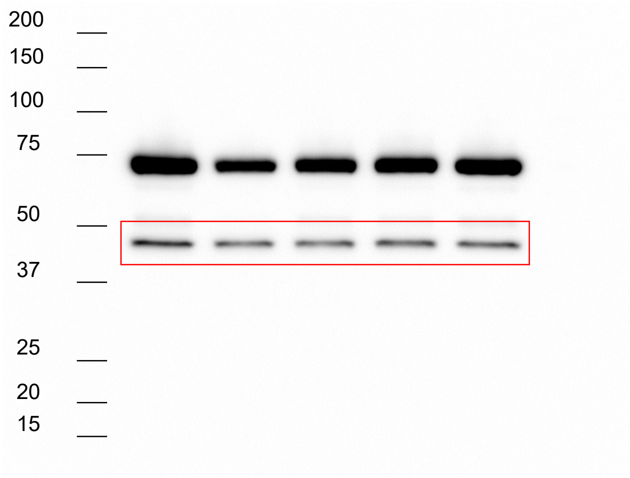

Supplement: SourceData F2 — is the source file for Fig. 2. [file jcb_202406097_sourcedataf2.pdf]
