# Supplementary material for: Dominant-negative isoform of TDP-43 is regulated by ALS-linked RNA-binding proteins
Source: J Cell Biol. 2025 Aug 8;224(10):e202406097. doi: 10.1083/jcb.202406097 (PMC12333503; doi:10.1083/jcb.202406097)

Source Data F5

D

TDP-43 (short exposure)

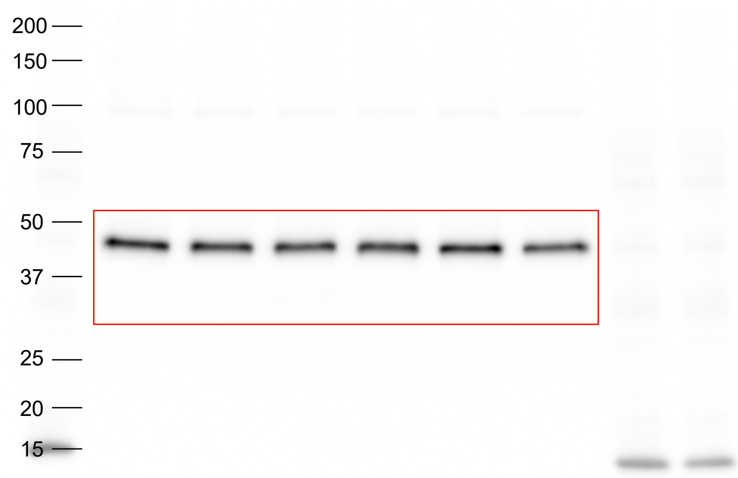

TDP-43 (long exposure)

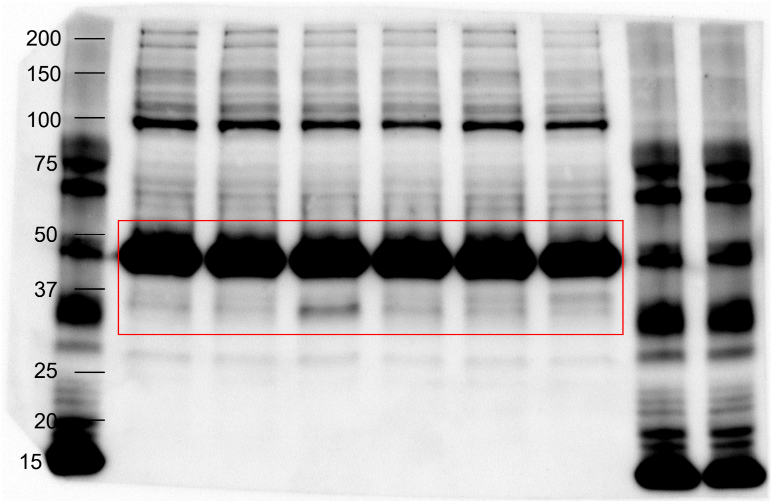

MP20

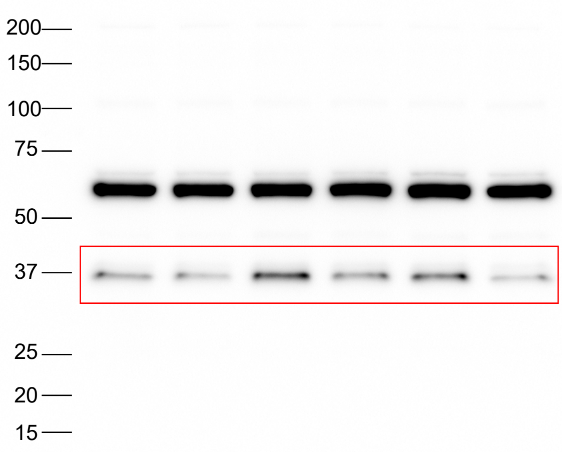

FLAG

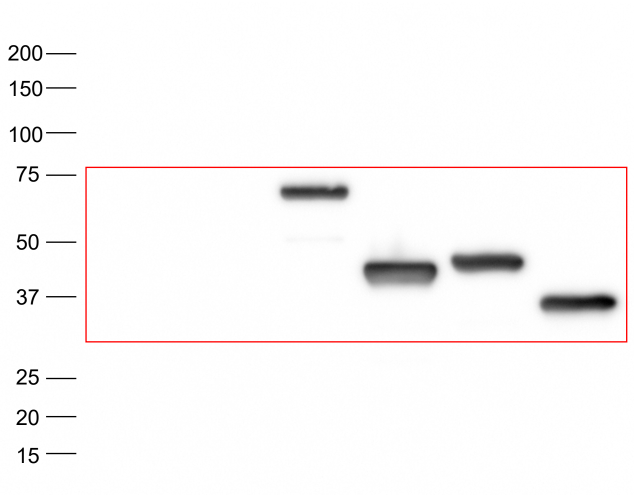

β-Actin
(reprobed following MP20 detection)

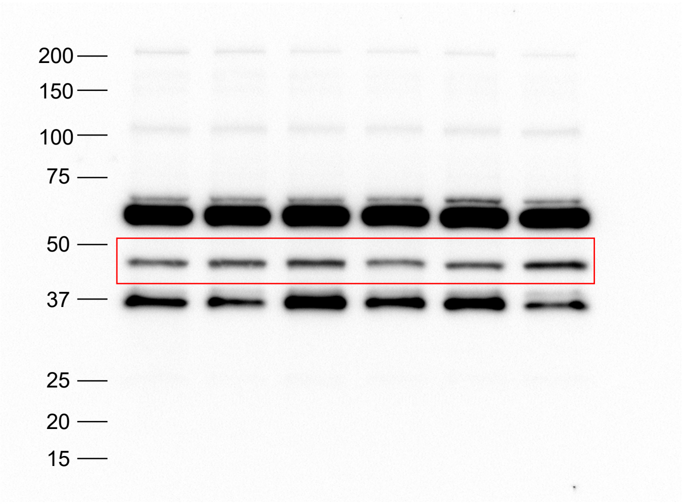

Source Data F5

E

TDP-43

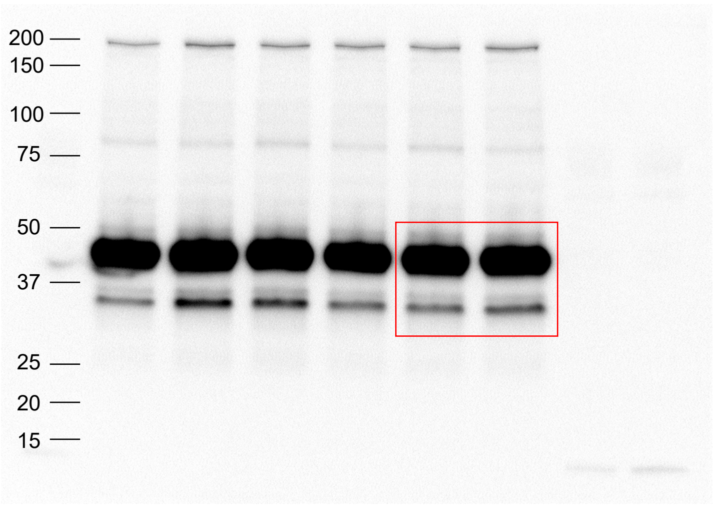

MP20

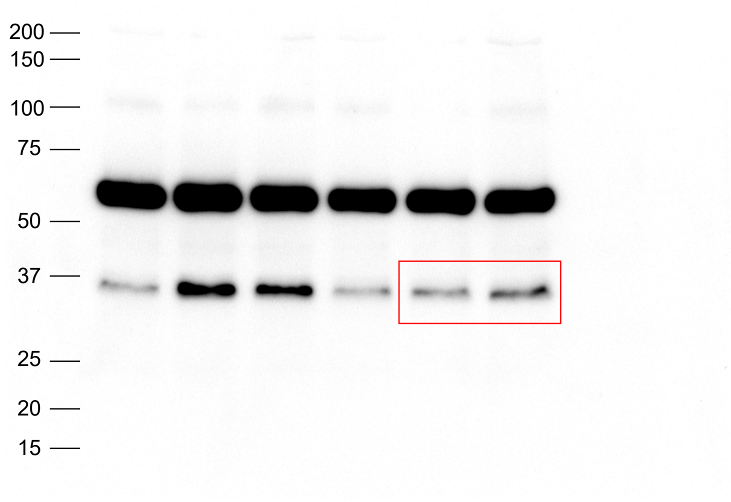

hnRNP K

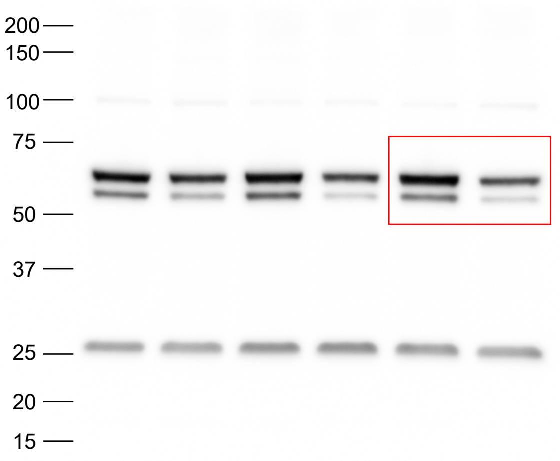

β-Actin
(reprobed following MP20 detection)

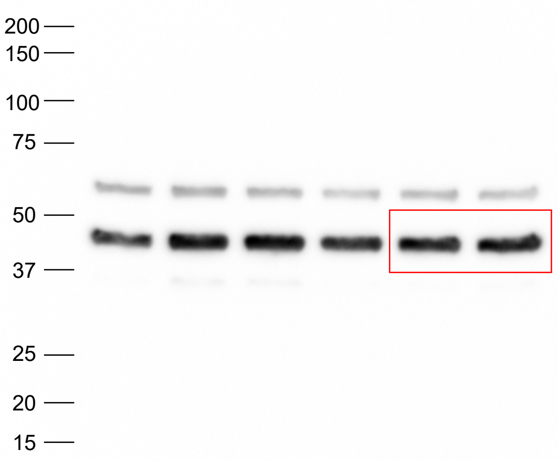

Source Data F5

F

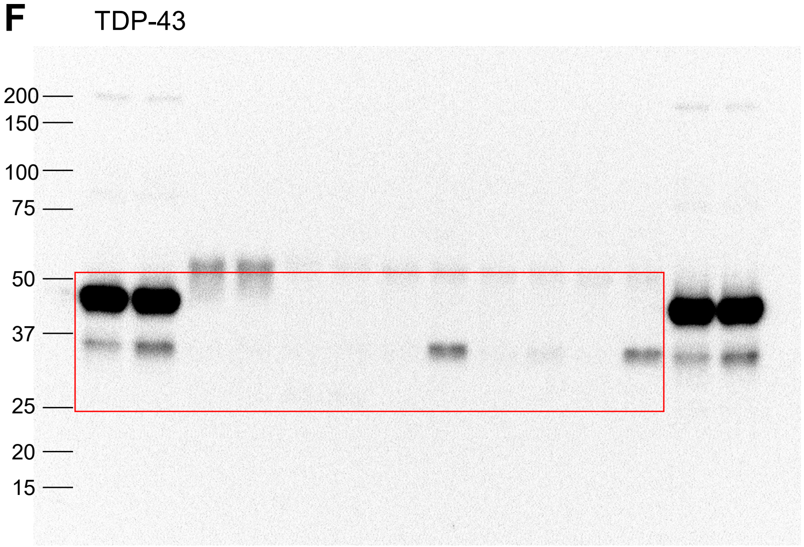

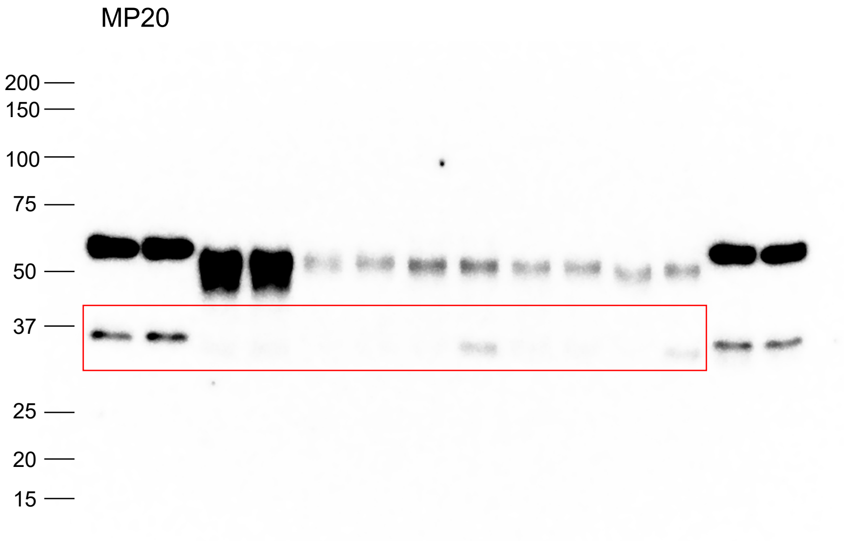

Source Data F5

F

FLAG

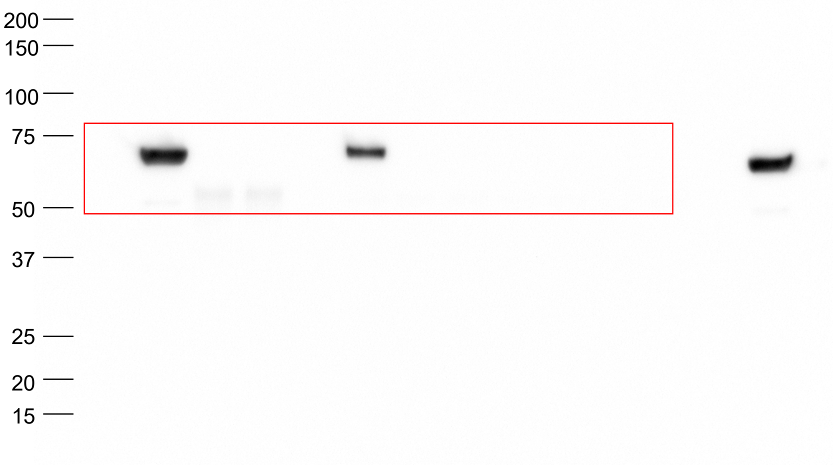

β-Actin
(reprobed following MP20 detection)

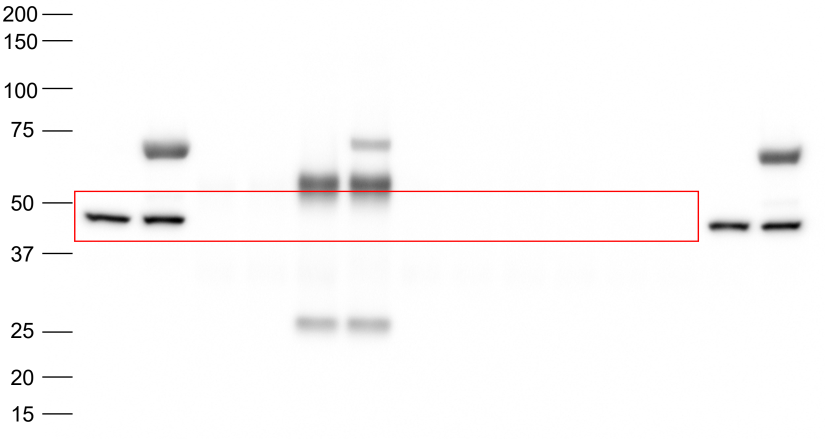

Supplement: SourceData F5 — is the source file for Fig. 5. [file jcb_202406097_sourcedataf5.pdf]
