# Supplementary material for: Dominant-negative isoform of TDP-43 is regulated by ALS-linked RNA-binding proteins
Source: J Cell Biol. 2025 Aug 8;224(10):e202406097. doi: 10.1083/jcb.202406097 (PMC12333503; doi:10.1083/jcb.202406097)

Source Data F6

A

hnRNP A1

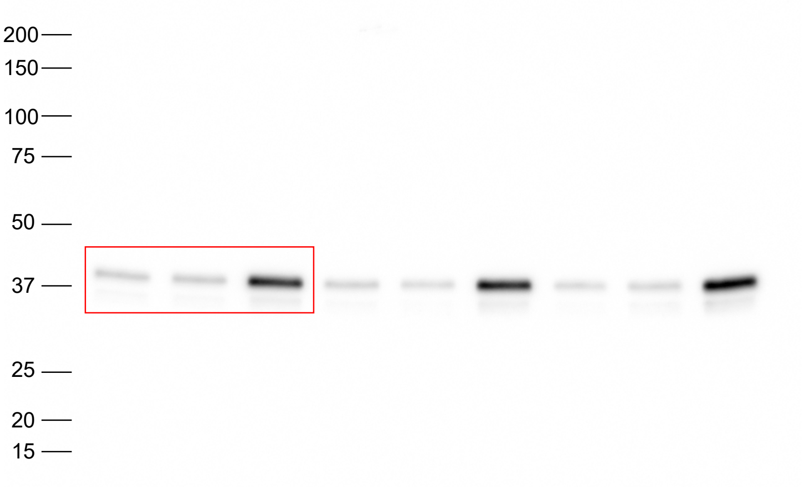

hnRNP A2/B1

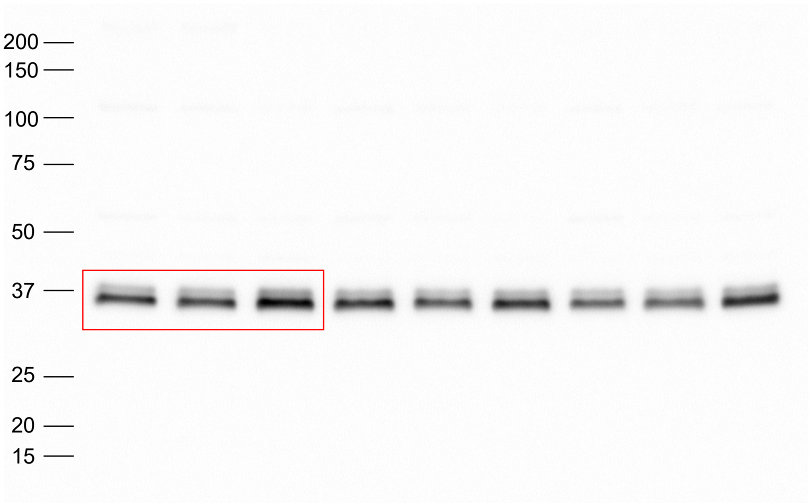

FLAG

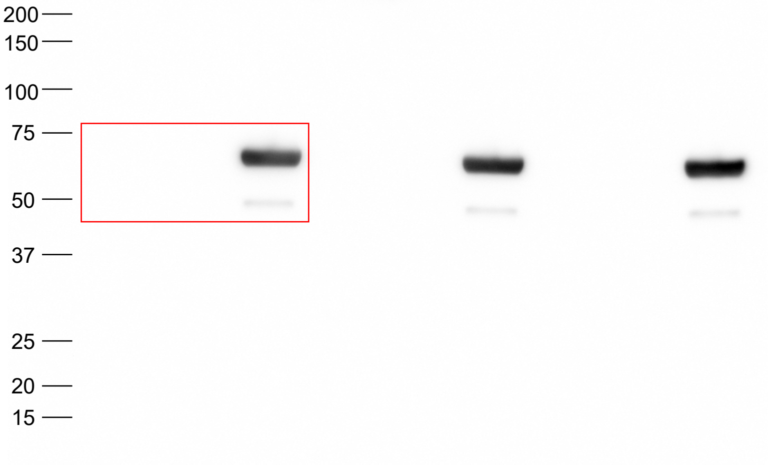

Source Data F6

A β-Actin
(reprobed following FLAG detection)

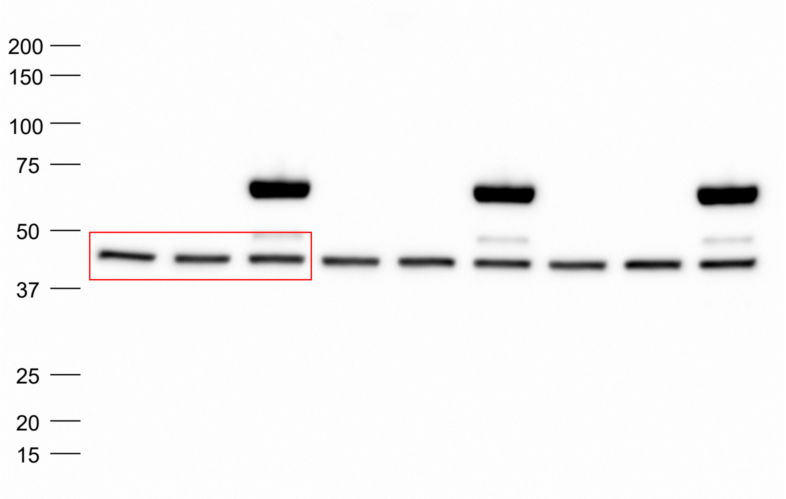

E hnRNP A1

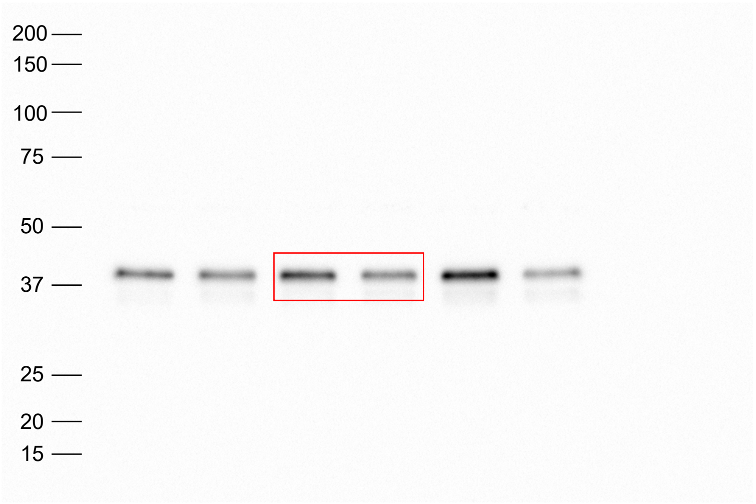

hnRNP K

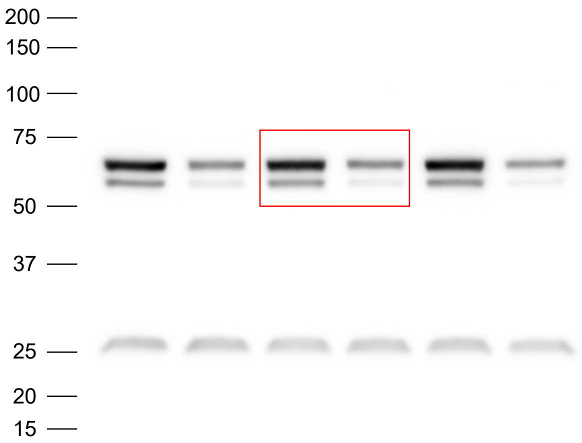

Source Data F6

E

β-Actin
(reprobed following FLAG detection)

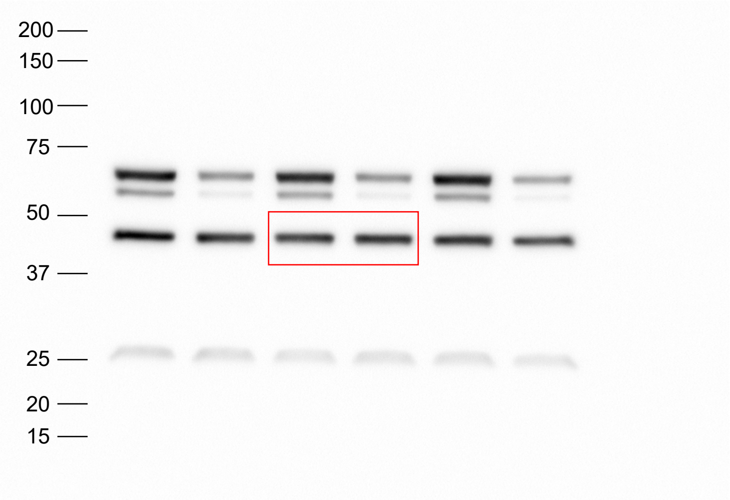

Supplement: SourceData F6 — is the source file for Fig. 6. [file jcb_202406097_sourcedataf6.pdf]
