# Supplementary material for: Dominant-negative isoform of TDP-43 is regulated by ALS-linked RNA-binding proteins
Source: J Cell Biol. 2025 Aug 8;224(10):e202406097. doi: 10.1083/jcb.202406097 (PMC12333503; doi:10.1083/jcb.202406097)

Source Data F7

B

Mini-gene

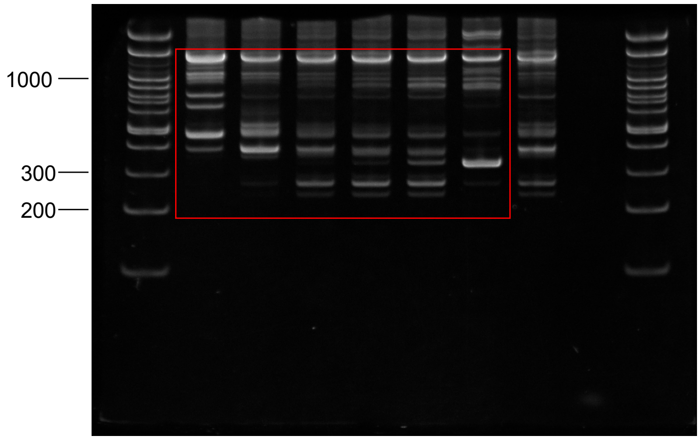

hnRNP K

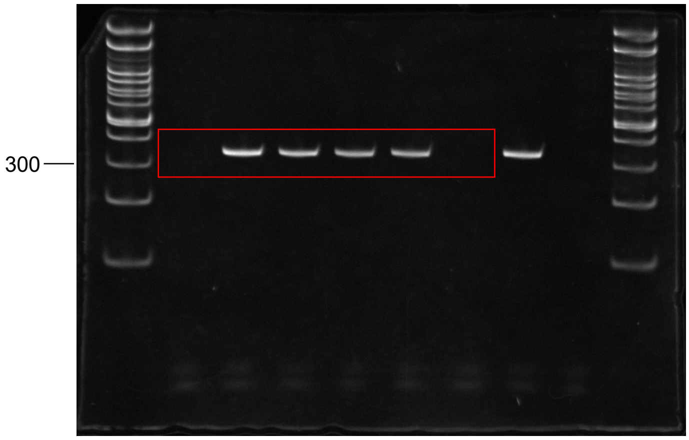

hnRNP A1

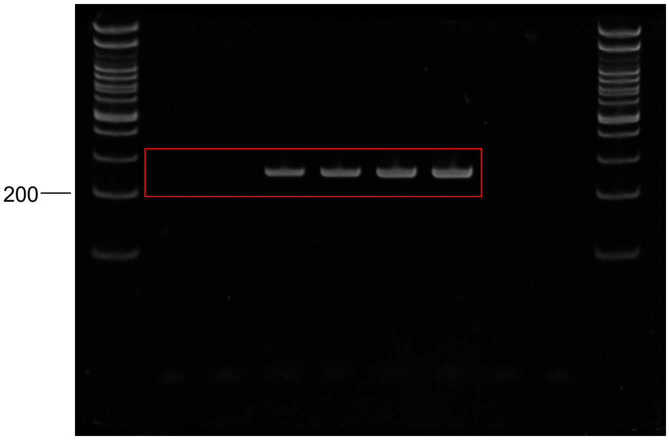

ACTB

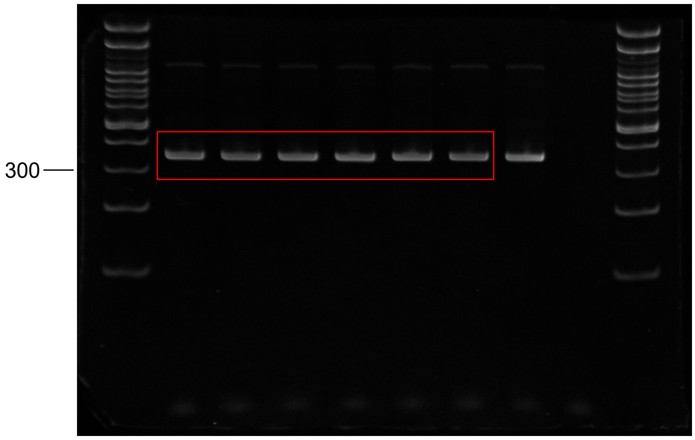

C

TDP-43 exon 5-7

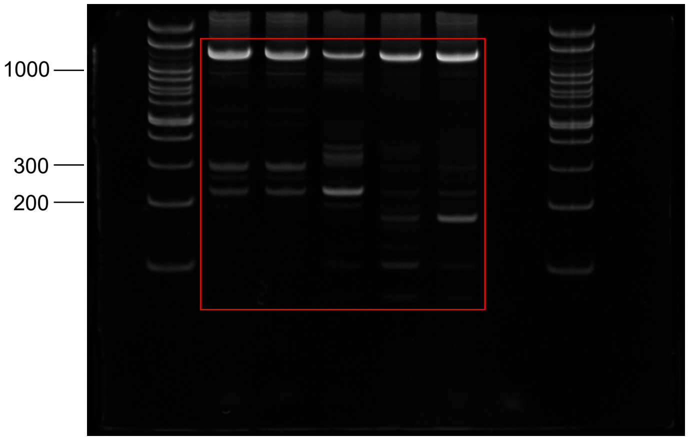

hnRNP K

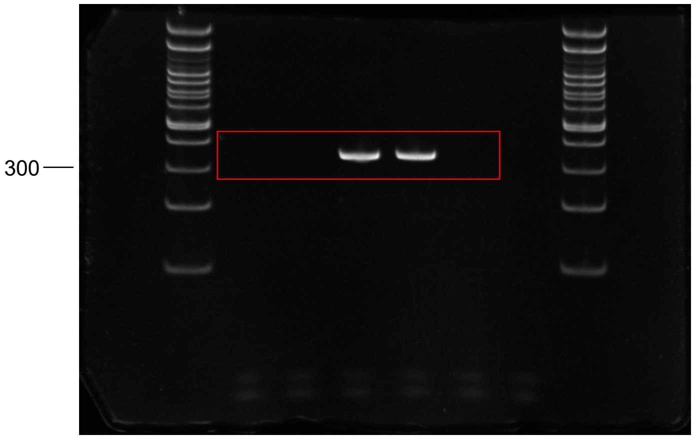

Source Data F7

C

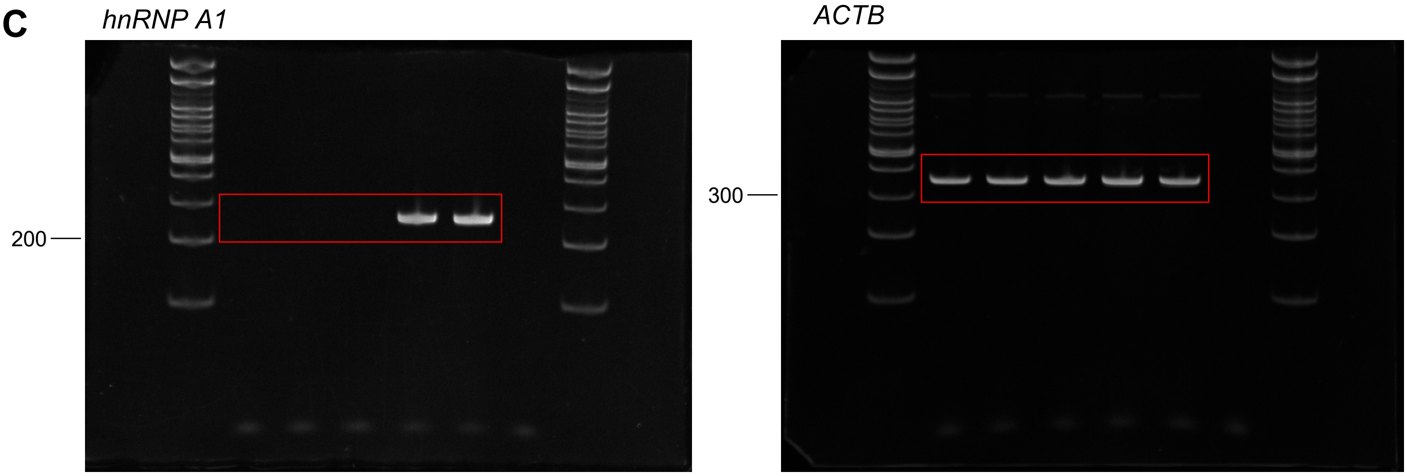

E

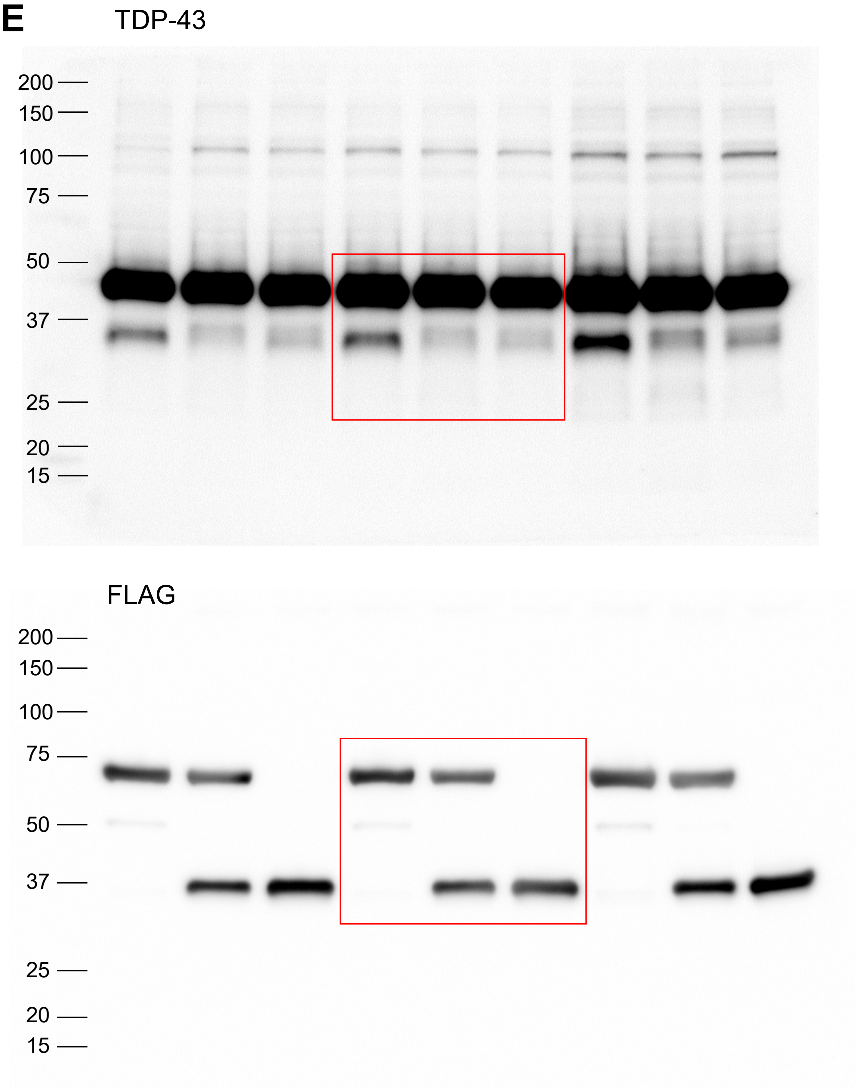

Source Data F7

E MP20

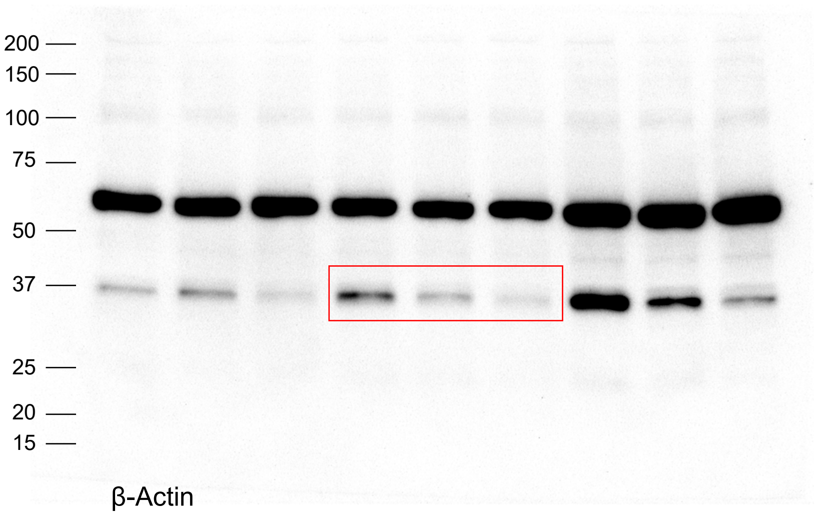

β-Actin
(reprobed following hnRNP K detection
(not described in this study))

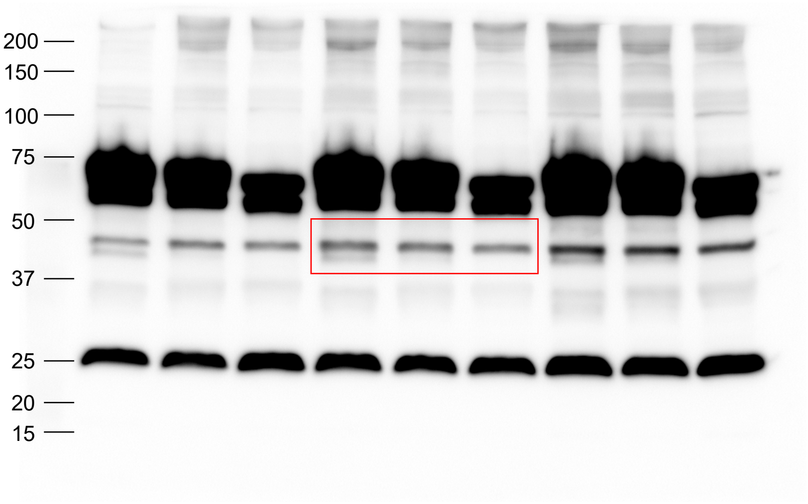

G *TDP-43 exon 5-7*

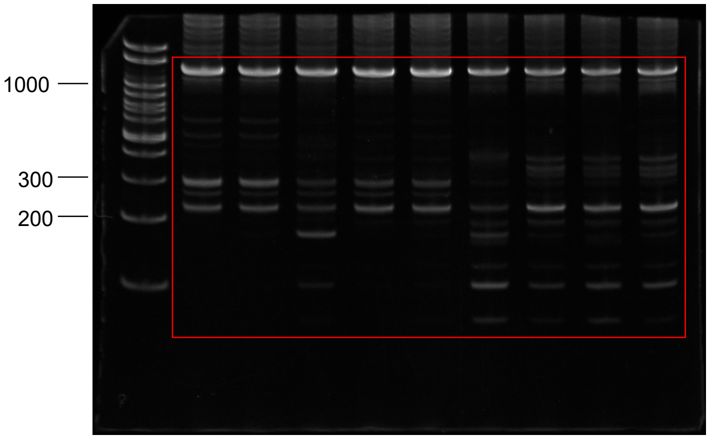

hnRNP K

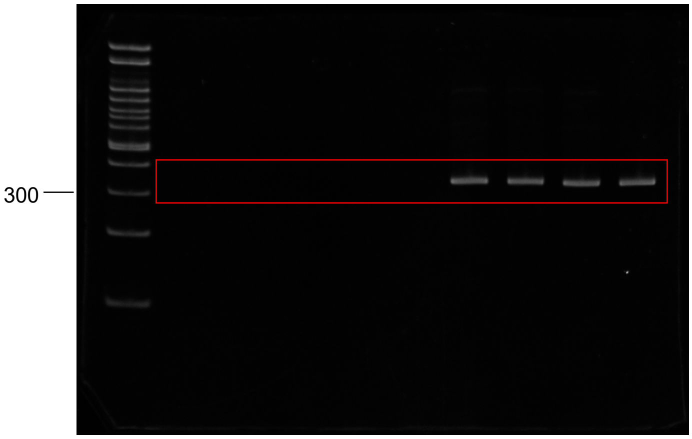

Source Data F7

G

hnRNP A1

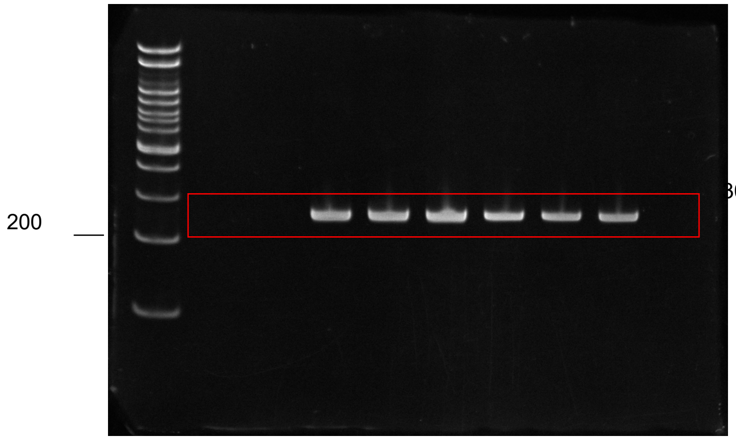

ACTB

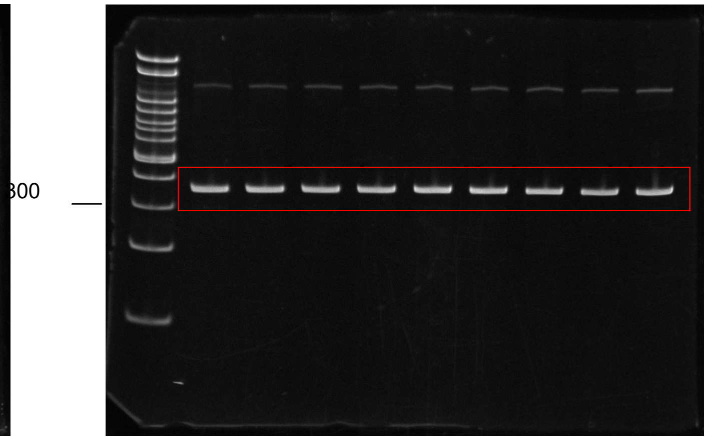

I

TDP-43

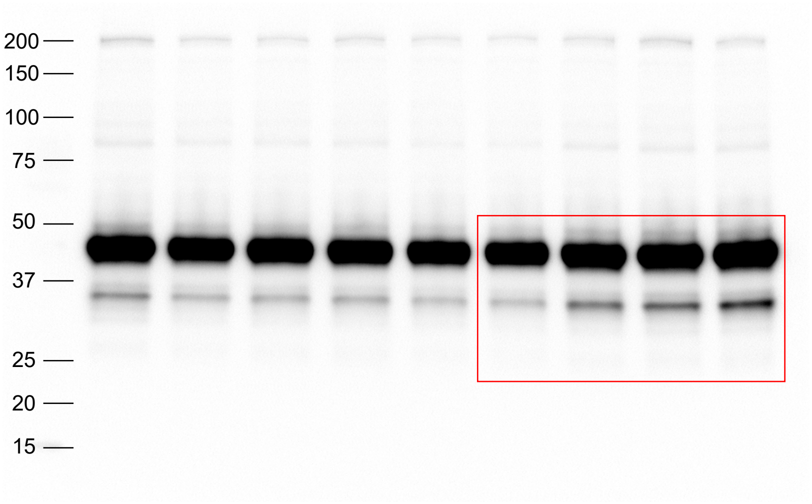

MP20

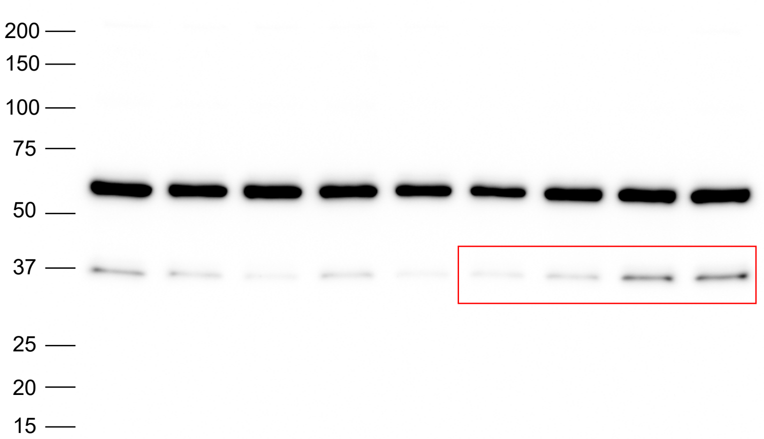

Source Data F7

I

FLAG

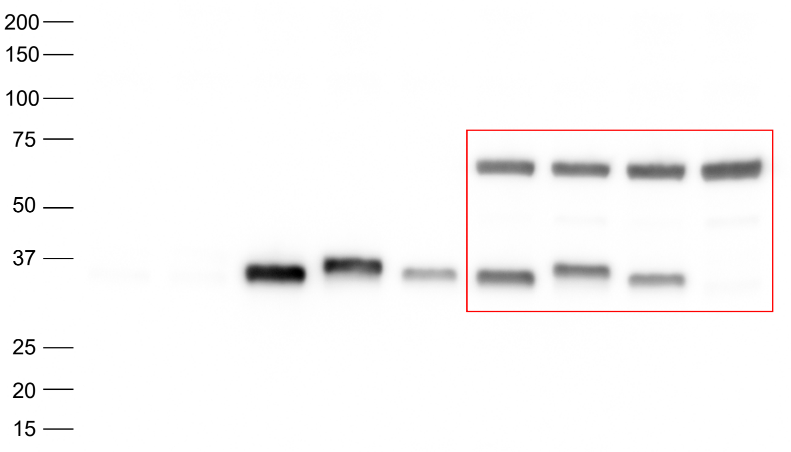

β-Actin

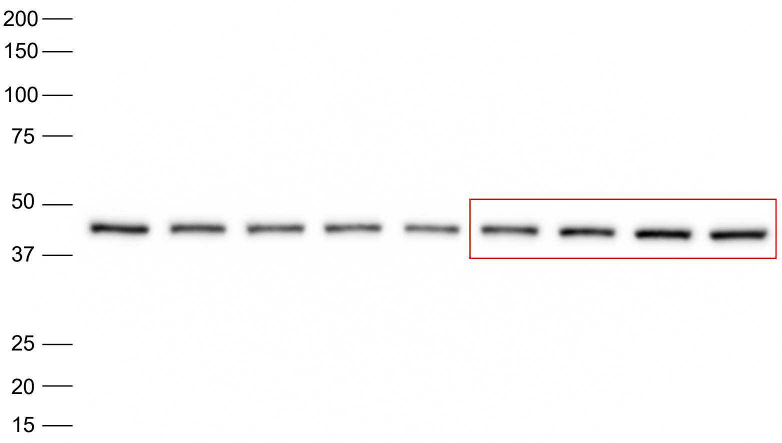

Supplement: SourceData F7 — is the source file for Fig. 7. [file jcb_202406097_sourcedataf7.pdf]
