# Supplementary material for: Dominant-negative isoform of TDP-43 is regulated by ALS-linked RNA-binding proteins
Source: J Cell Biol. 2025 Aug 8;224(10):e202406097. doi: 10.1083/jcb.202406097 (PMC12333503; doi:10.1083/jcb.202406097)

Source Data F8

B

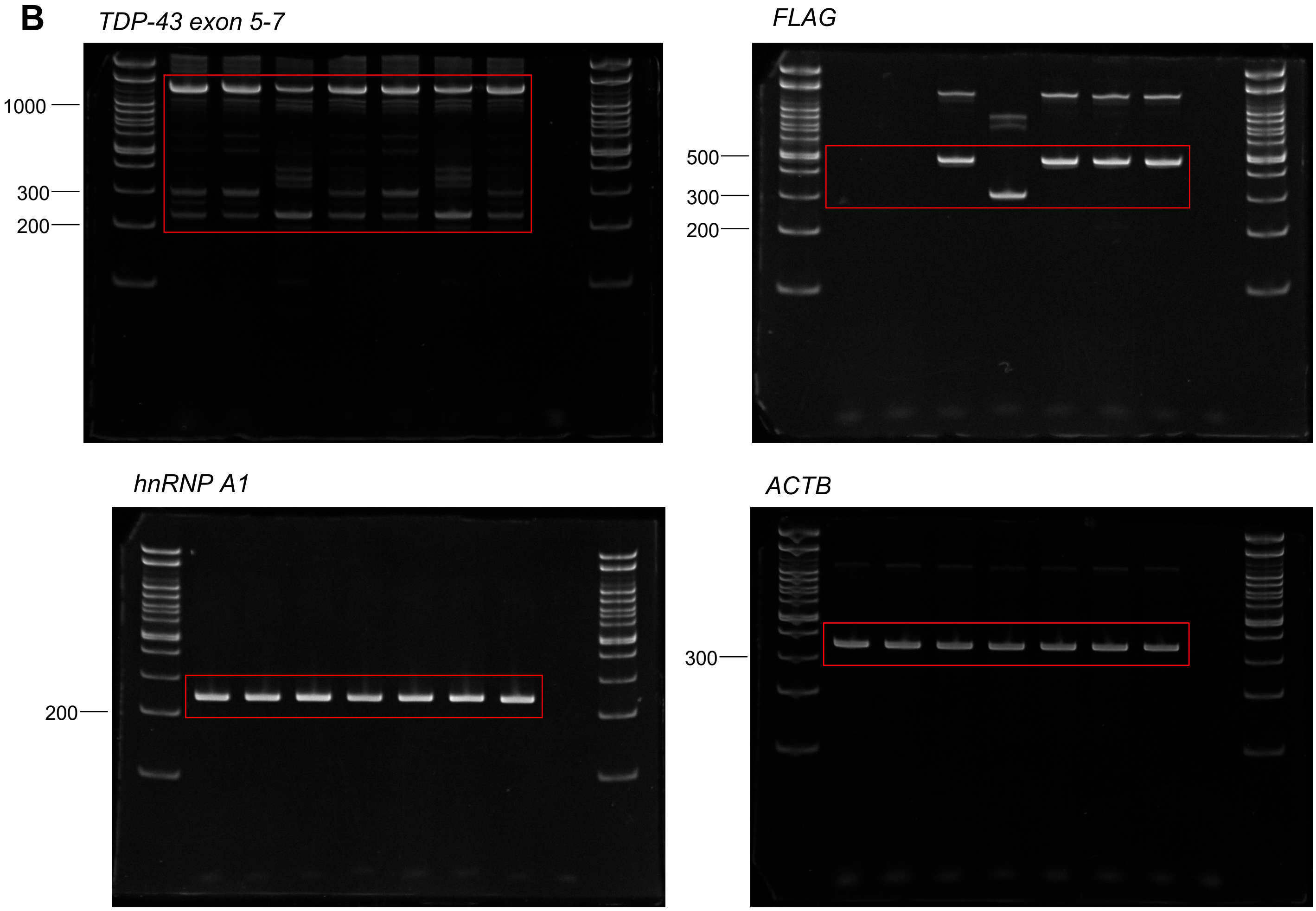

D

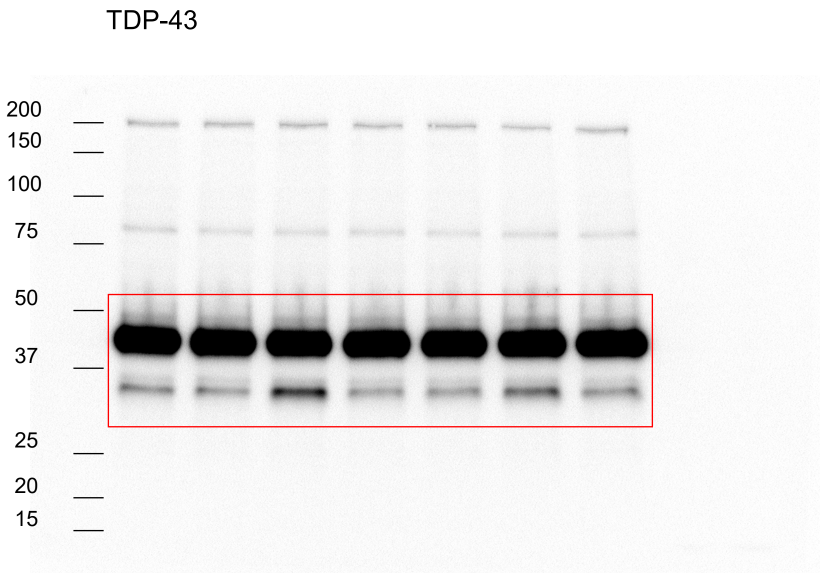

Source Data F8

D MP20

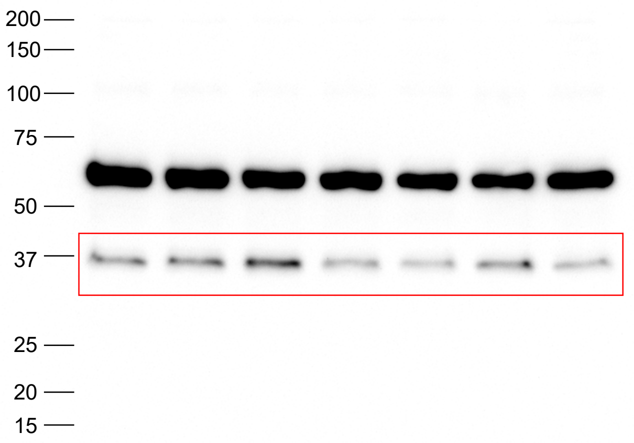

hnRNP A1

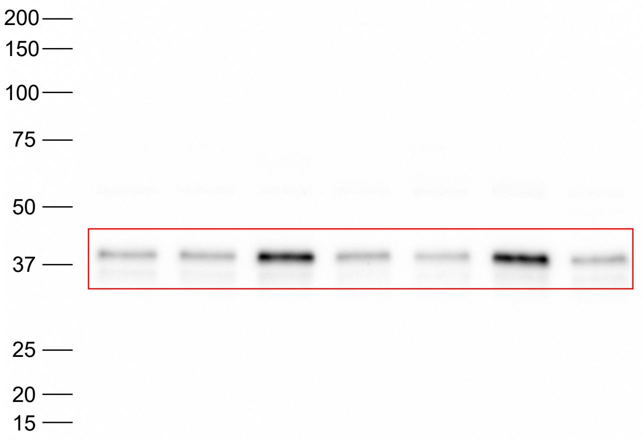

hnRNP A2/B1

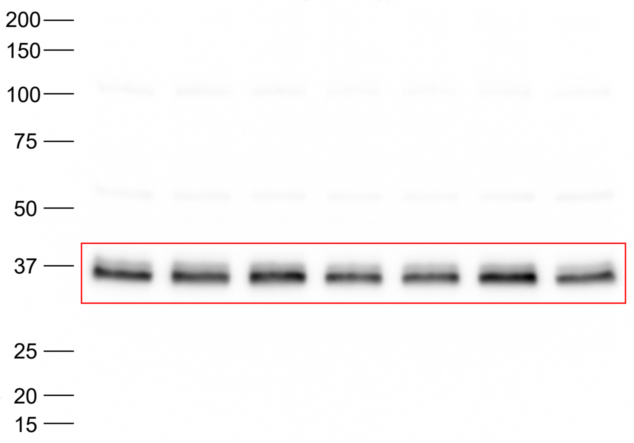

Source Data F8

D FLAG
(reprobed following TDP-43 detection)

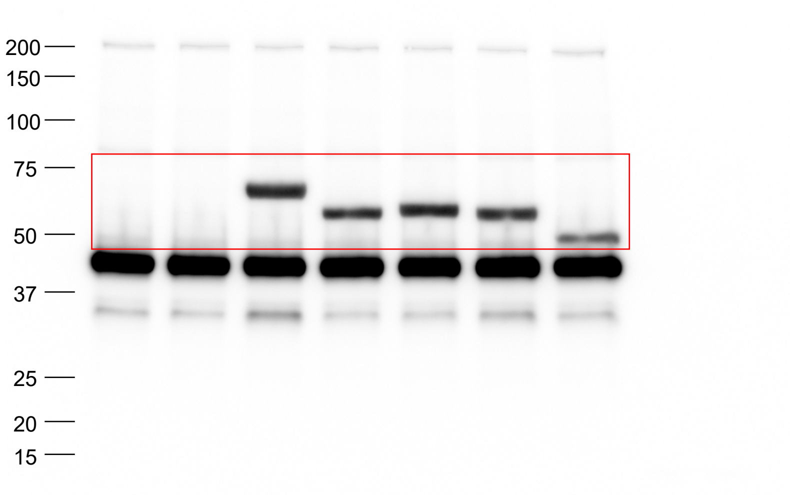

β-Actin
(reprobed following hnRNP A1 detection)

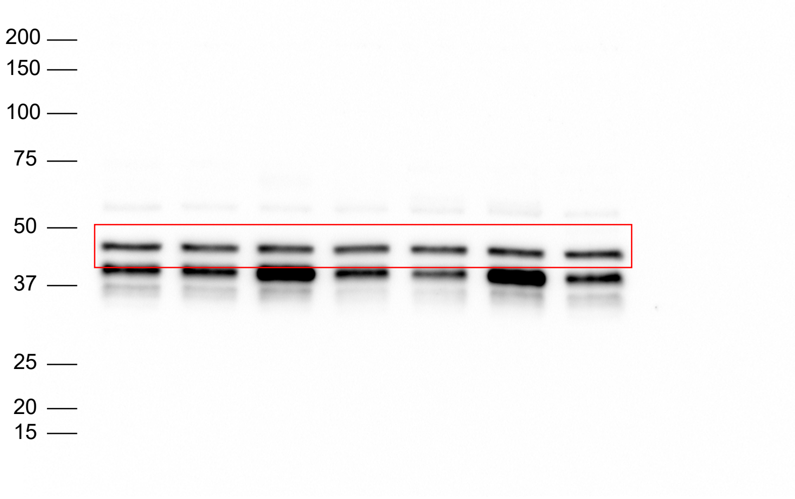

Source Data F8

E

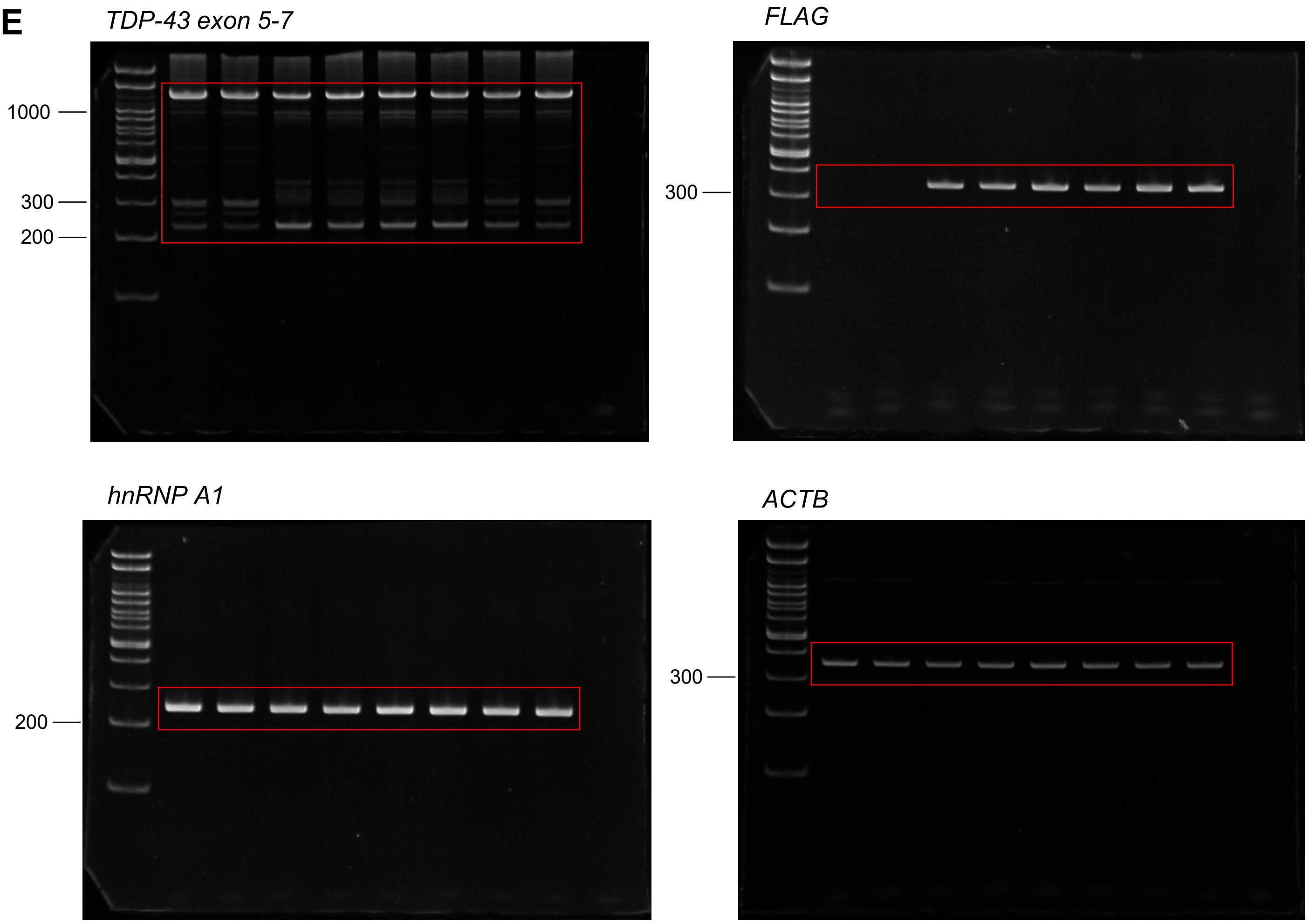

G

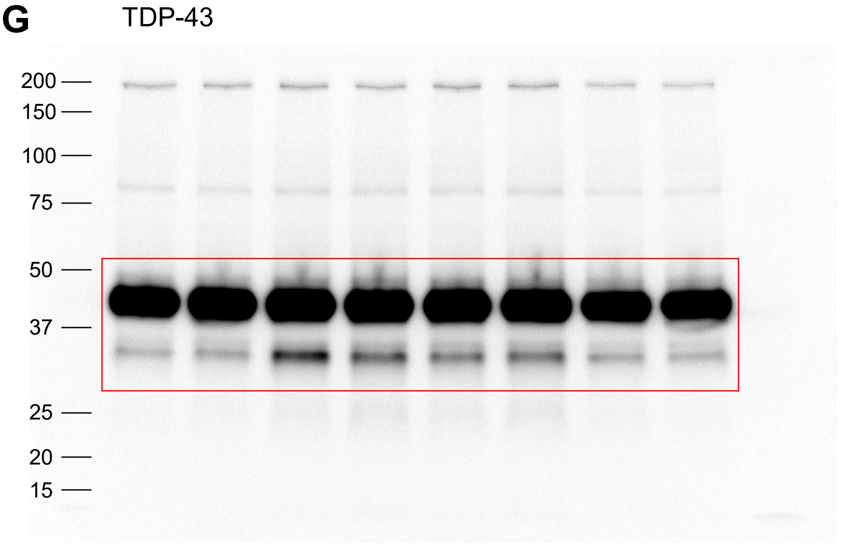

Source Data F8

G MP20

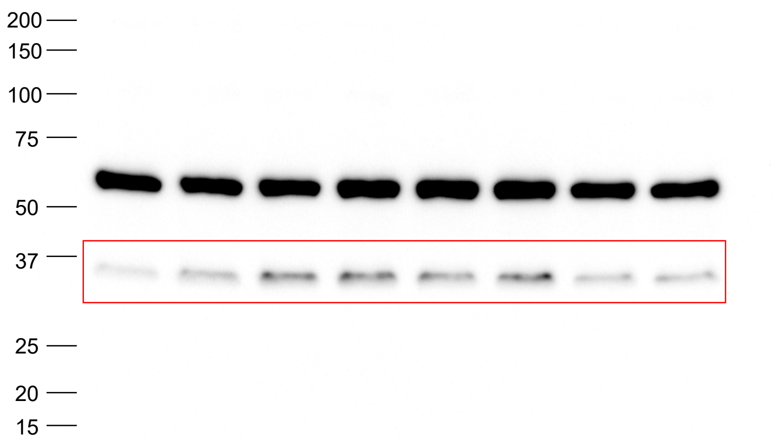

hnRNP A1

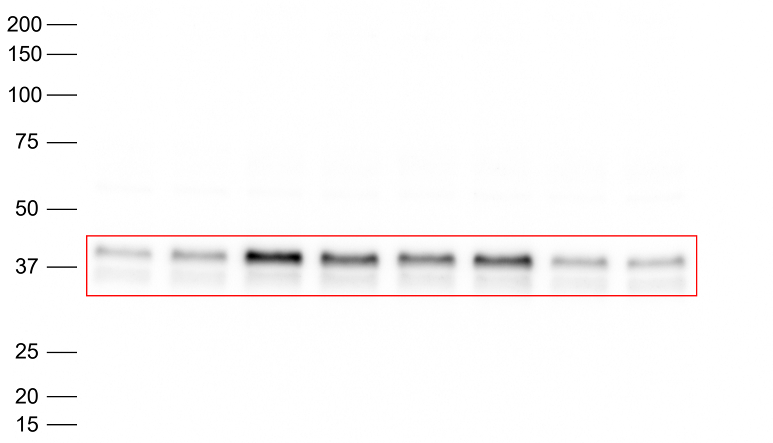

hnRNP A2/B1

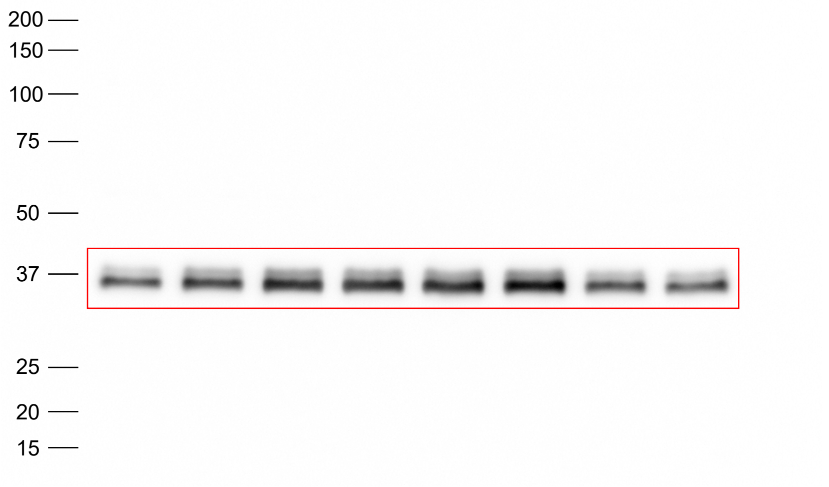

Source Data F8

G FLAG

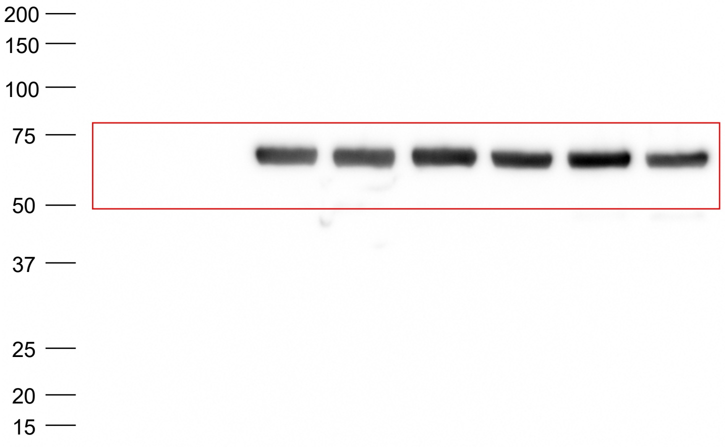

β-Actin
(reprobed following FLAG detection)

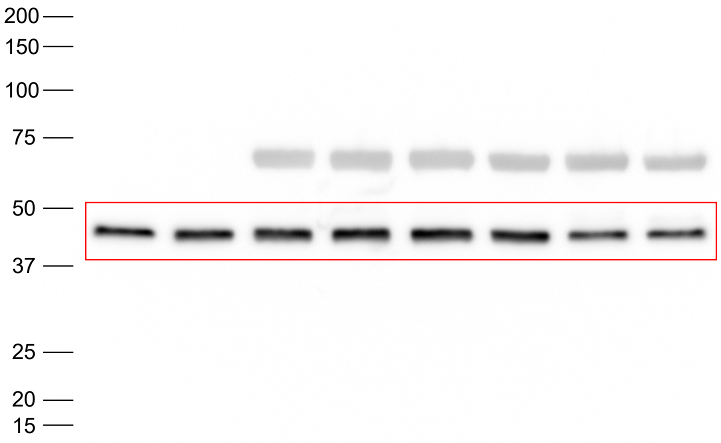

Supplement: SourceData F8 — is the source file for Fig. 8. [file jcb_202406097_sourcedataf8.pdf]
