# Supplementary material for: Dominant-negative isoform of TDP-43 is regulated by ALS-linked RNA-binding proteins
Source: J Cell Biol. 2025 Aug 8;224(10):e202406097. doi: 10.1083/jcb.202406097 (PMC12333503; doi:10.1083/jcb.202406097)

Source Data F9

D hnRNP K

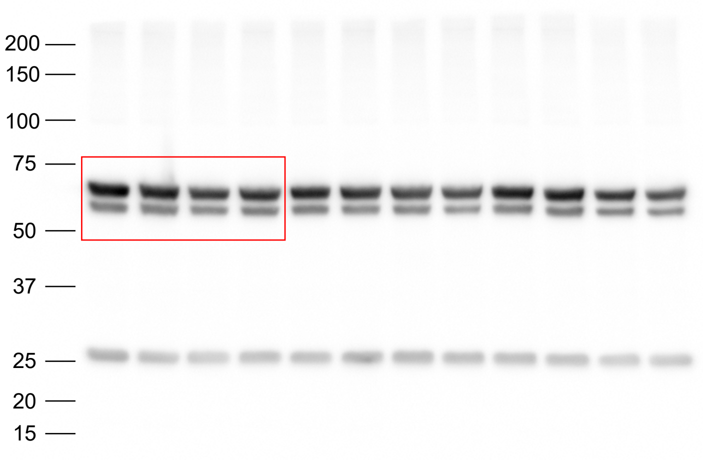

FLAG
(reprobed following β-Actin detection)

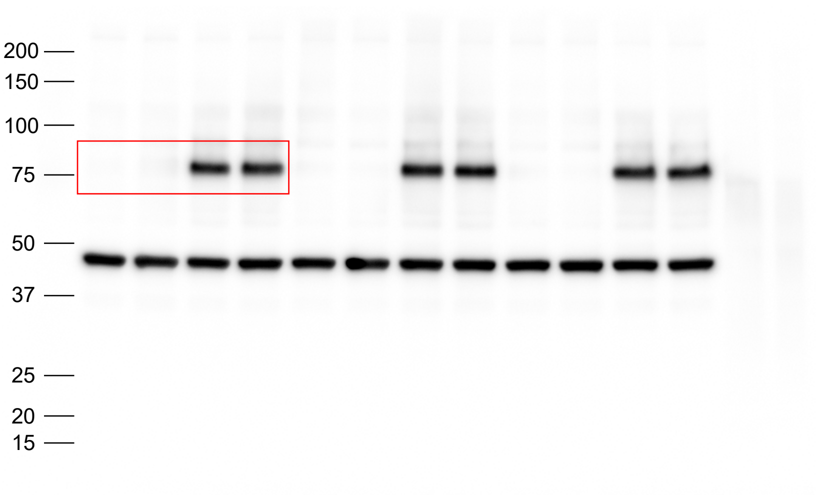

β-Actin

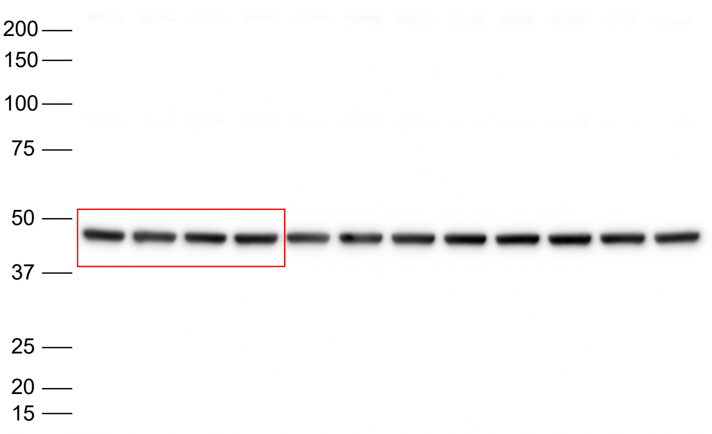

Source Data F9

F MP20

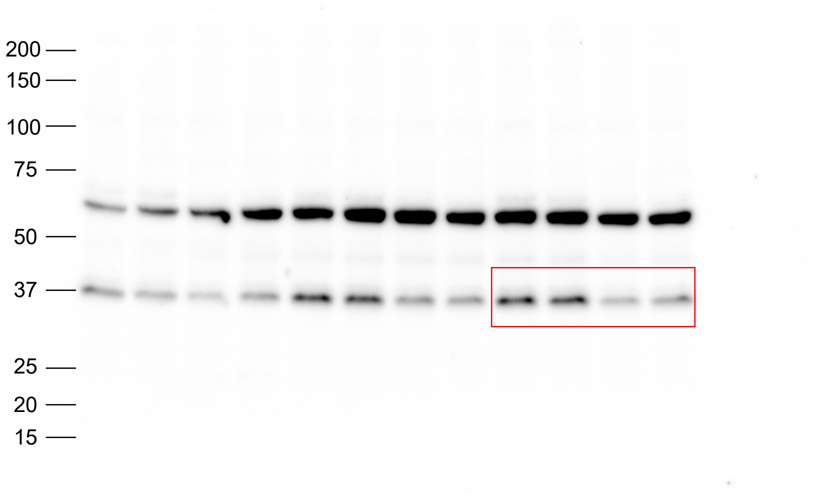

TDP-43 (short exposure)

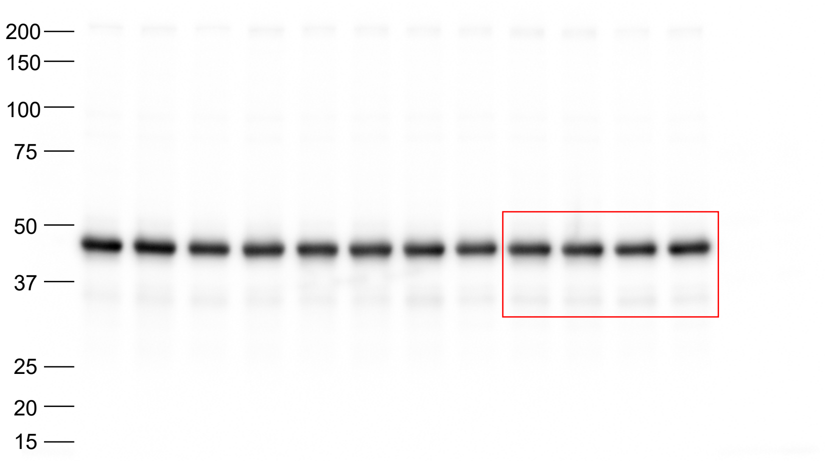

TDP-43 (long exposure)

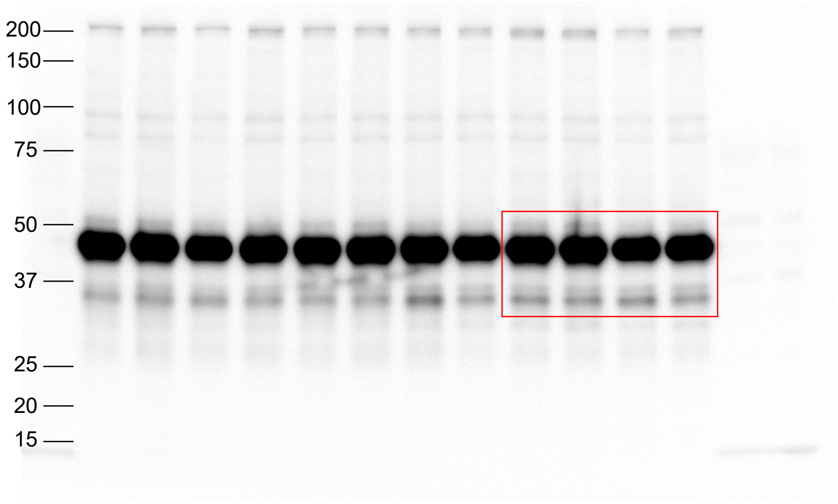

Source Data F9

F β-Actin

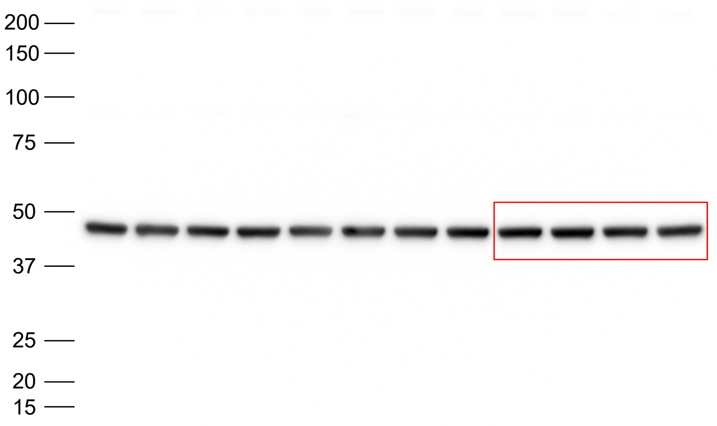

FLAG
(reprobed following β-Actin detection)

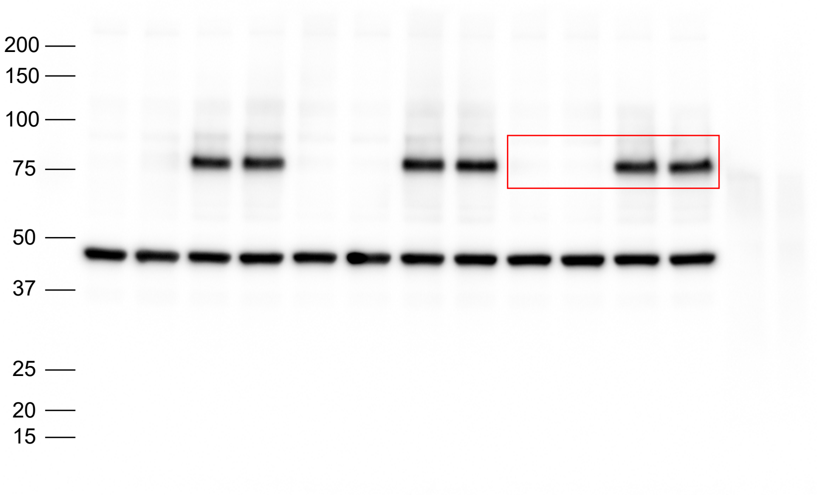

Supplement: SourceData F9 — is the source file for Fig. 9. [file jcb_202406097_sourcedataf9.pdf]
