# Supplementary material for: Dominant-negative isoform of TDP-43 is regulated by ALS-linked RNA-binding proteins
Source: J Cell Biol. 2025 Aug 8;224(10):e202406097. doi: 10.1083/jcb.202406097 (PMC12333503; doi:10.1083/jcb.202406097)

Source Data FS1

A FLAG

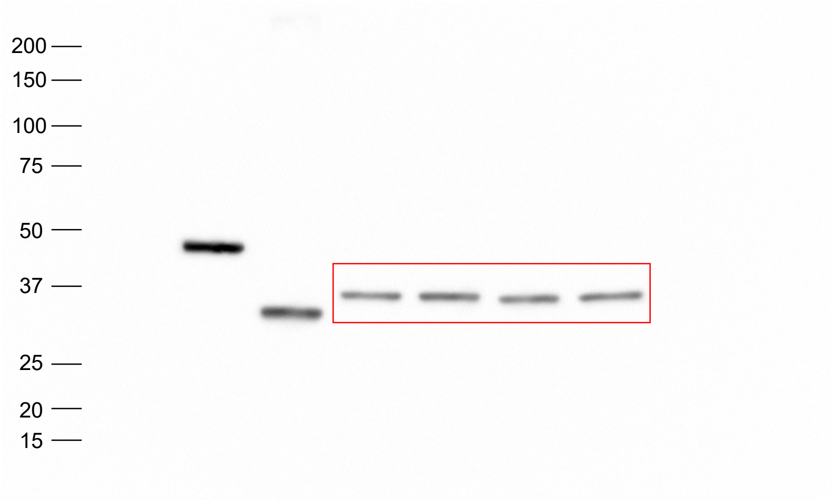

TDP-43

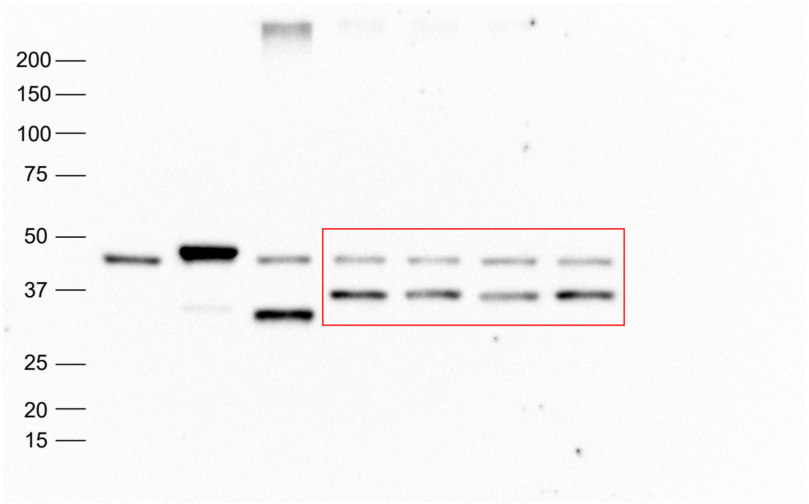

GAPDH

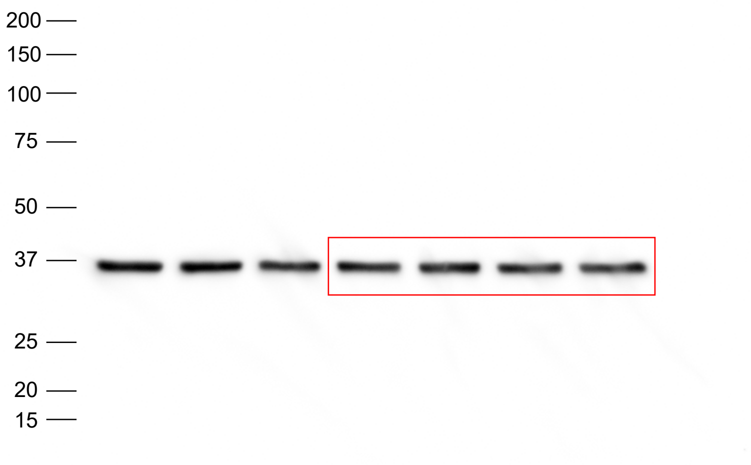

Source Data FS1

C TDP-43

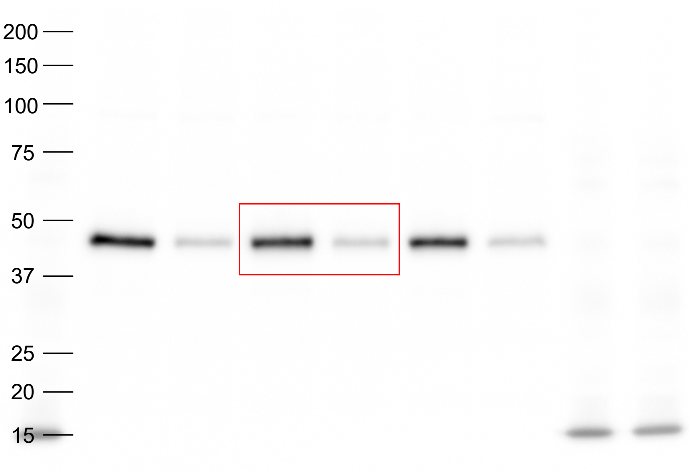

β-Actin

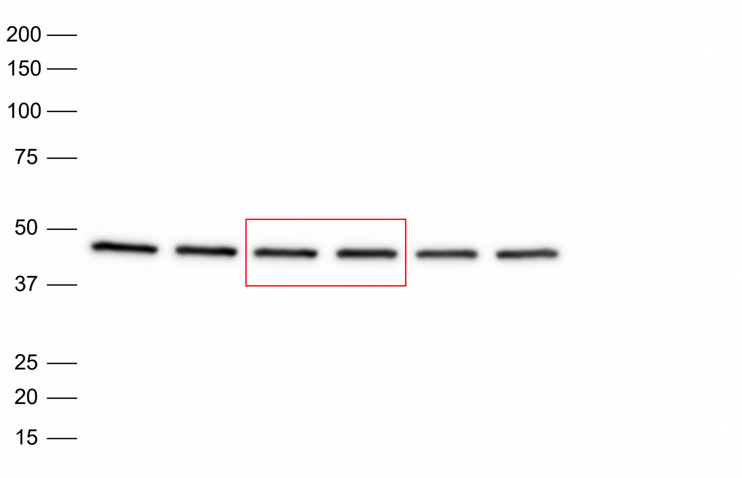

E FLAG

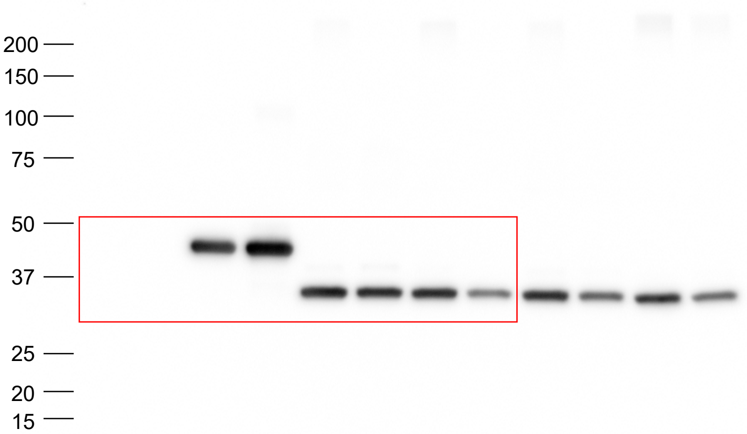

Source Data FS1

E

β-Actin

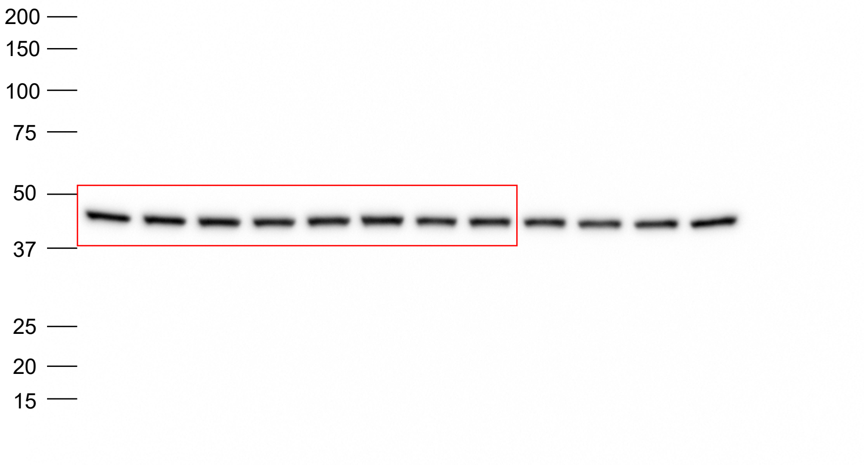

I

TDP-43

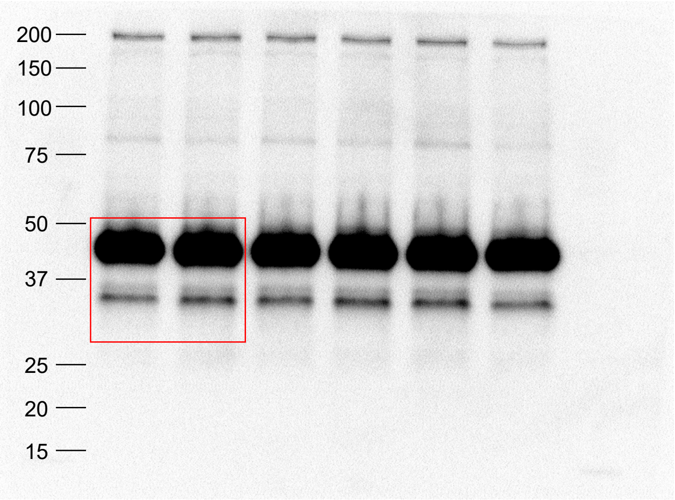

MP20

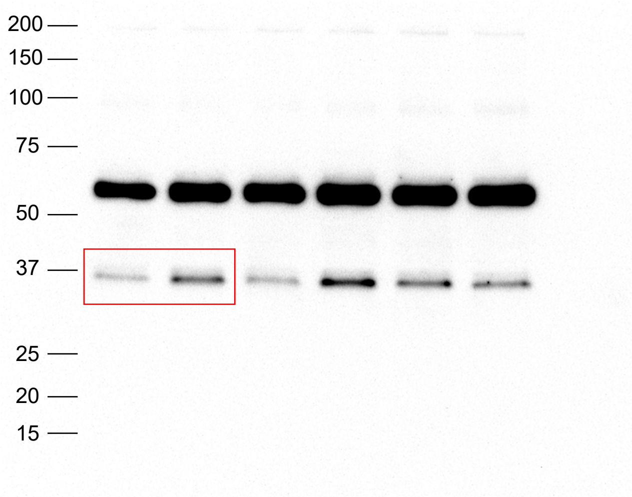

β-Actin
(reprobed following MP20 detection)

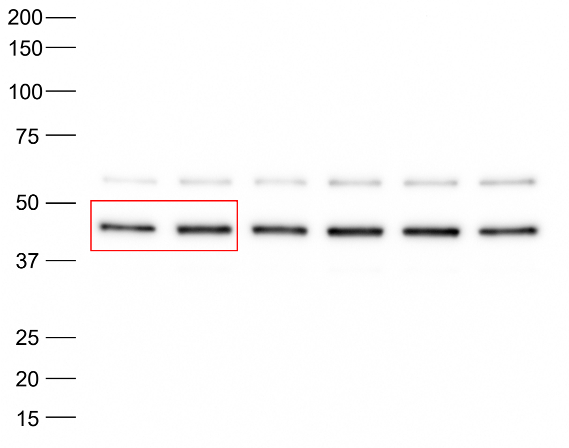

hnRNP A1

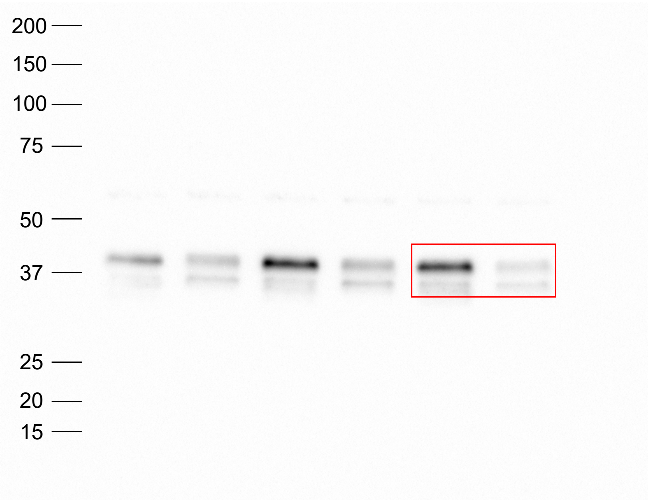

Supplement: SourceData FS1 — is the source file for Fig. S1. [file jcb_202406097_sourcedatafs1.pdf]
