# Supplementary material for: Dominant-negative isoform of TDP-43 is regulated by ALS-linked RNA-binding proteins
Source: J Cell Biol. 2025 Aug 8;224(10):e202406097. doi: 10.1083/jcb.202406097 (PMC12333503; doi:10.1083/jcb.202406097)

Source Data FS2

D hnRNP K

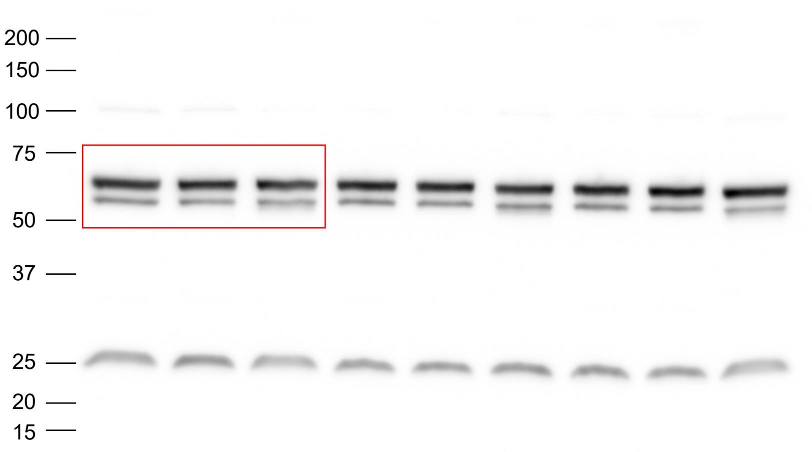

FLAG

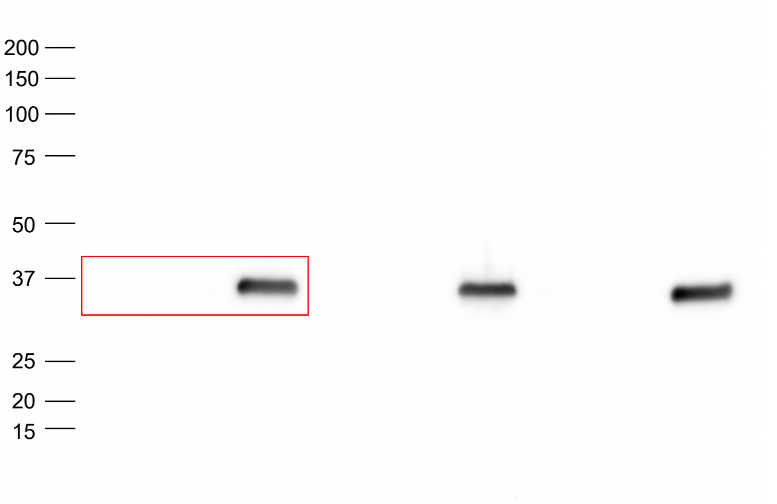

β-Actin
(reprobed following hnRNP K detection)

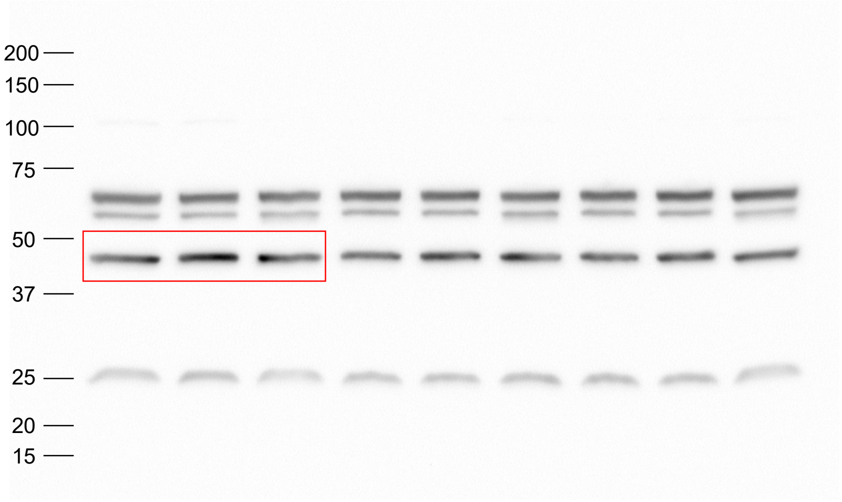

Source Data FS2

F

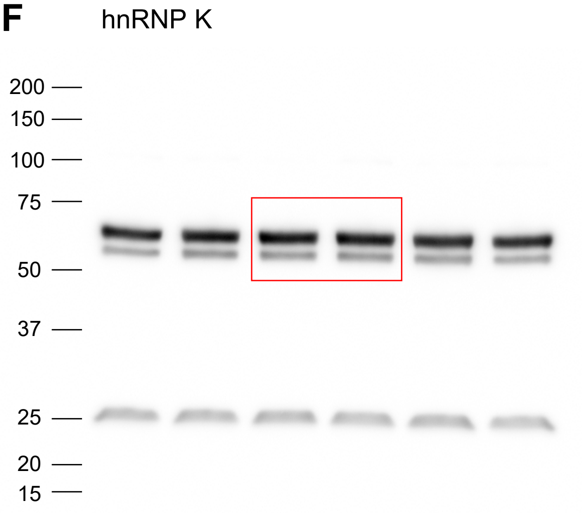

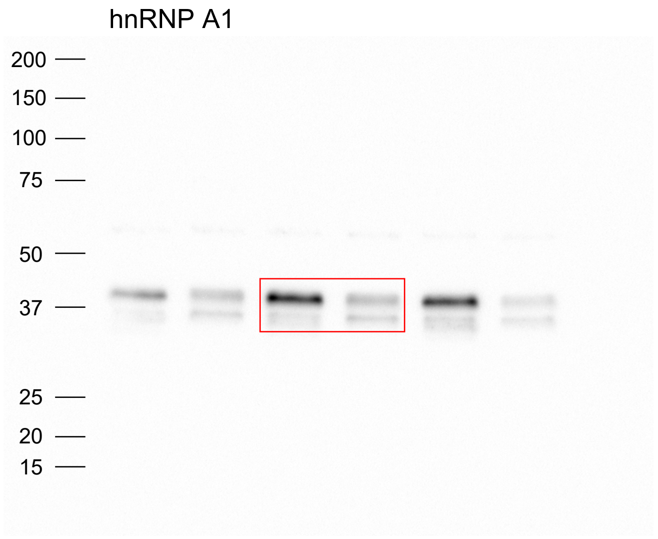

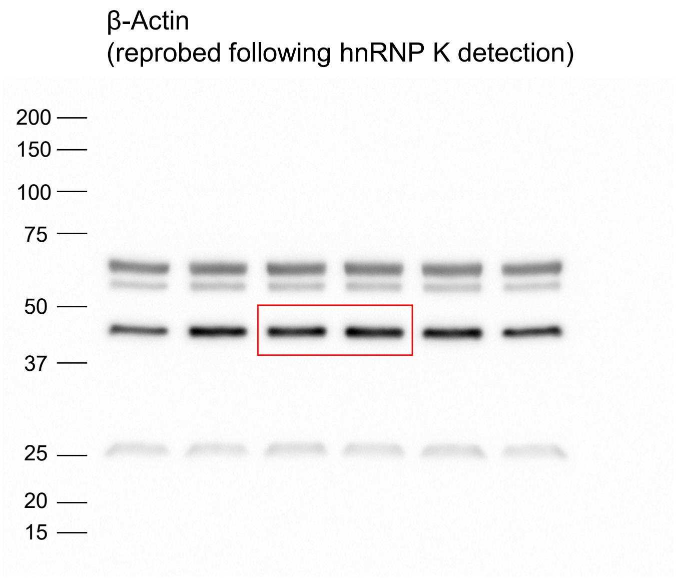

Source Data FS2

H

hnRNP K (low contrast)

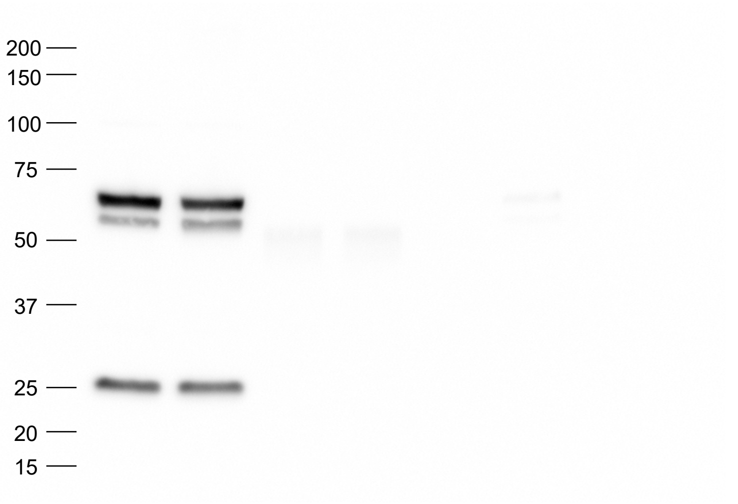

hnRNP K (high contrast)

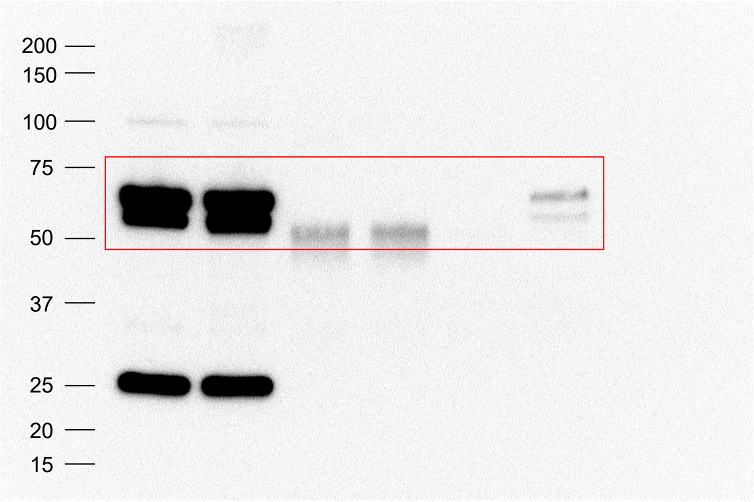

FLAG

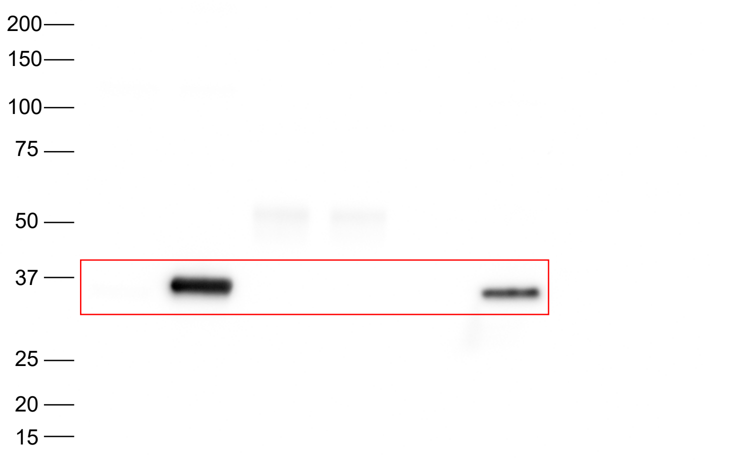

Source Data FS2

H β-Actin
(reprobed following hnRNP K detection)

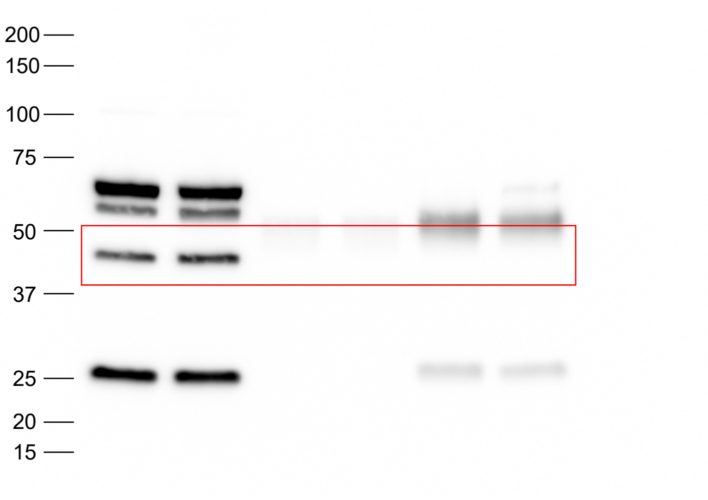

Supplement: SourceData FS2 — is the source file for Fig. S2. [file jcb_202406097_sourcedatafs2.pdf]
