# Supplementary material for: Dominant-negative isoform of TDP-43 is regulated by ALS-linked RNA-binding proteins
Source: J Cell Biol. 2025 Aug 8;224(10):e202406097. doi: 10.1083/jcb.202406097 (PMC12333503; doi:10.1083/jcb.202406097)

Source Data FS5

C *Ex6 410-566*

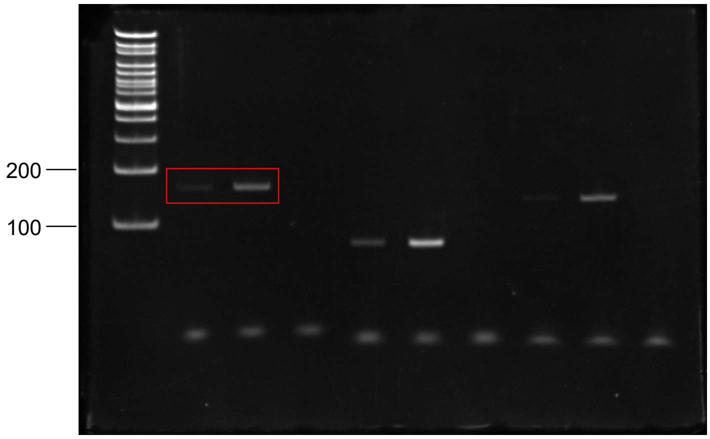

FUS
(low contrast)

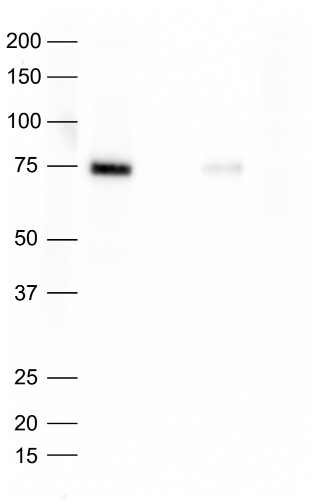

FUS
(high contrast)

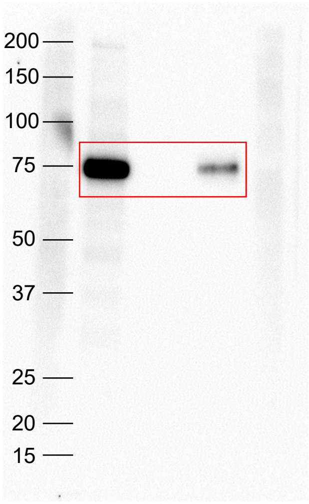

β-Actin
(reprobed following
FUS detection)

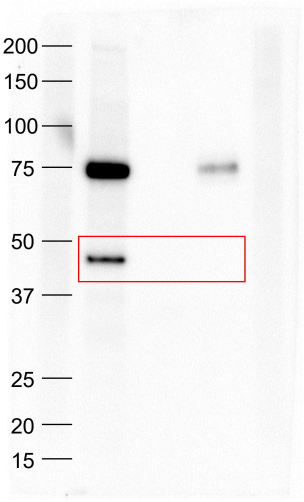

Source Data FS5

F Venus (Venus construct)

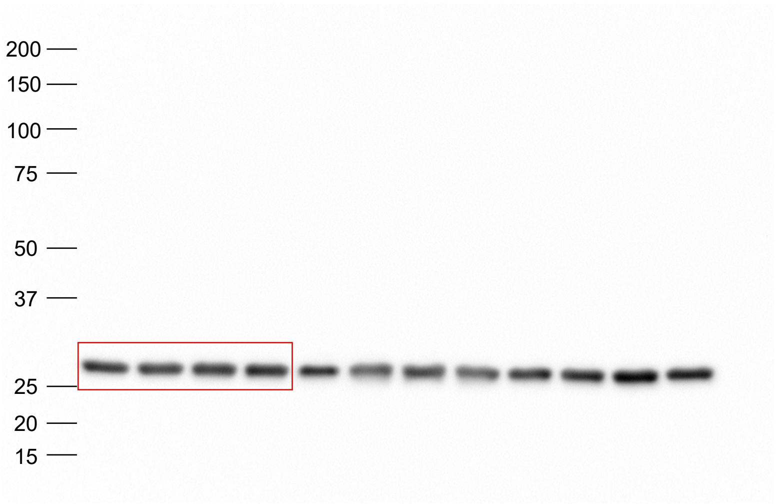

β-Actin
(reprobed following Venus detection)

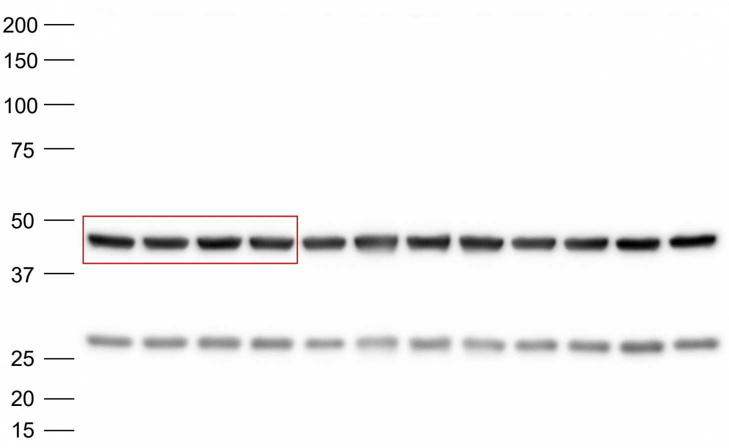

FLAG
(reprobed following Venus and β-Actin detection)

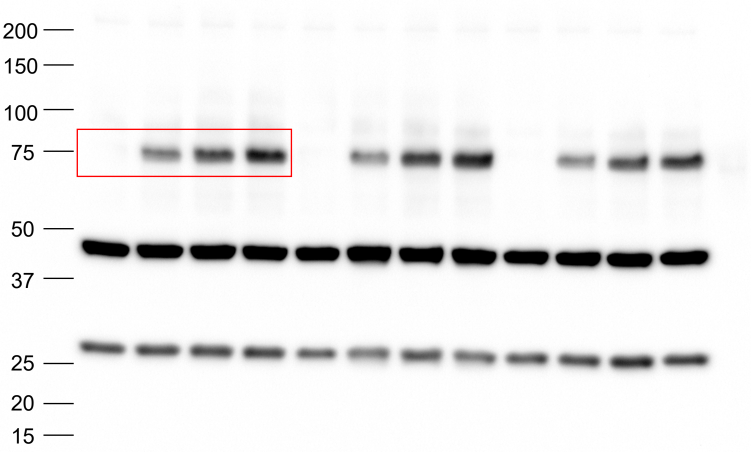

Source Data FS5

F Venus (Venus-3′UTR construct)

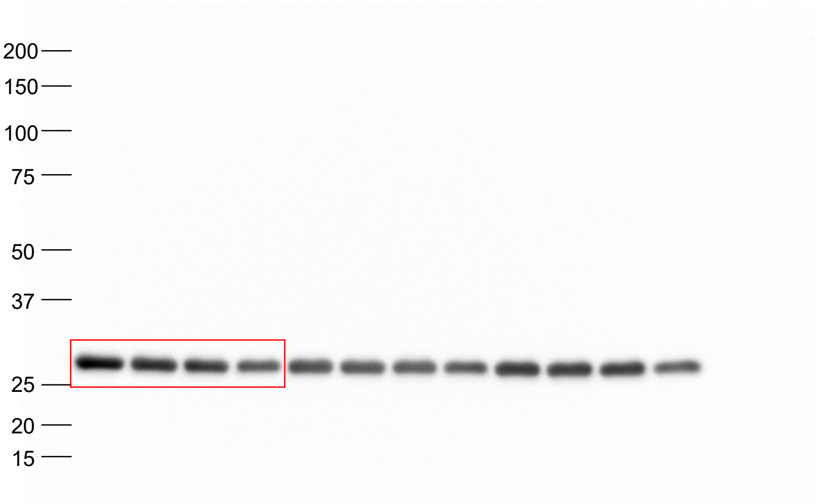

β-Actin
(reprobed following Venus detection)

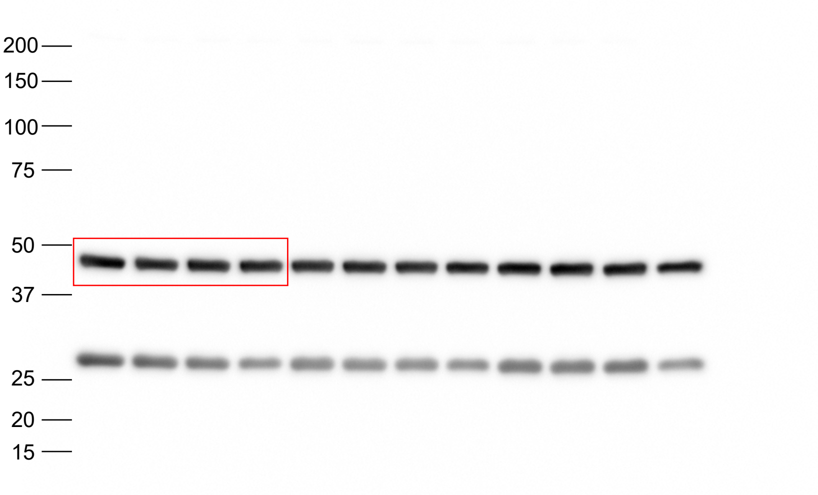

FLAG
(reprobed following Venus and β-Actin detection)

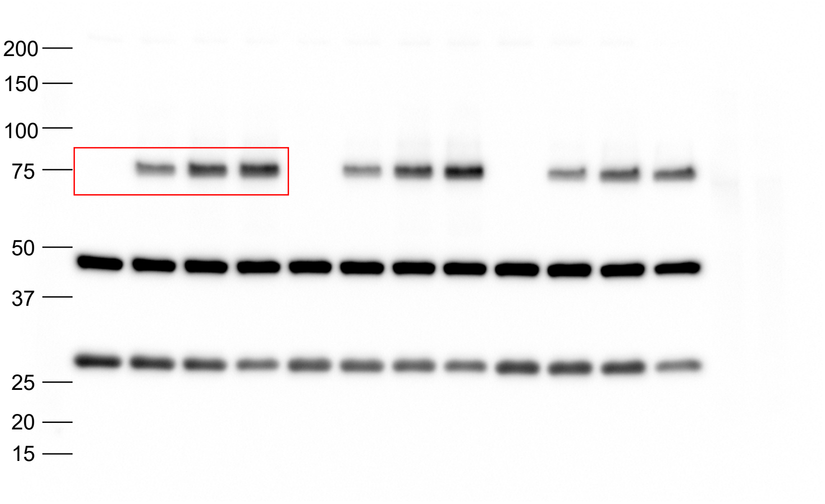

Source Data FS5

G

Venus (Venus construct)

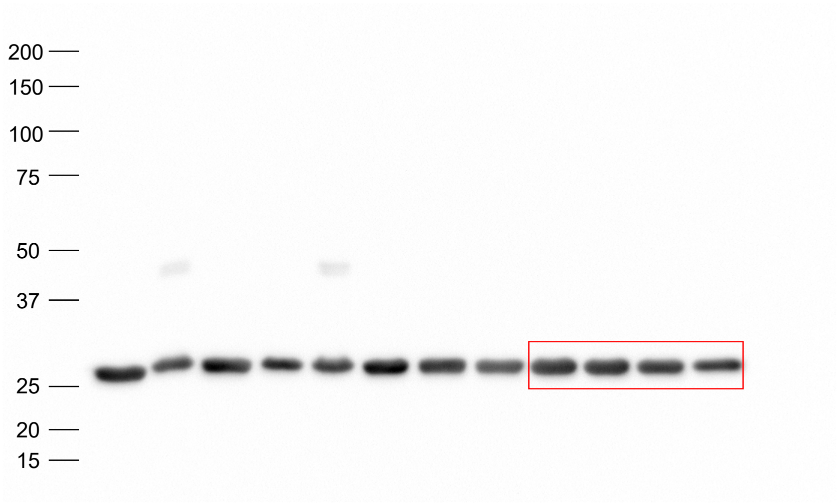

β-Actin
(reprobed following Venus detection)

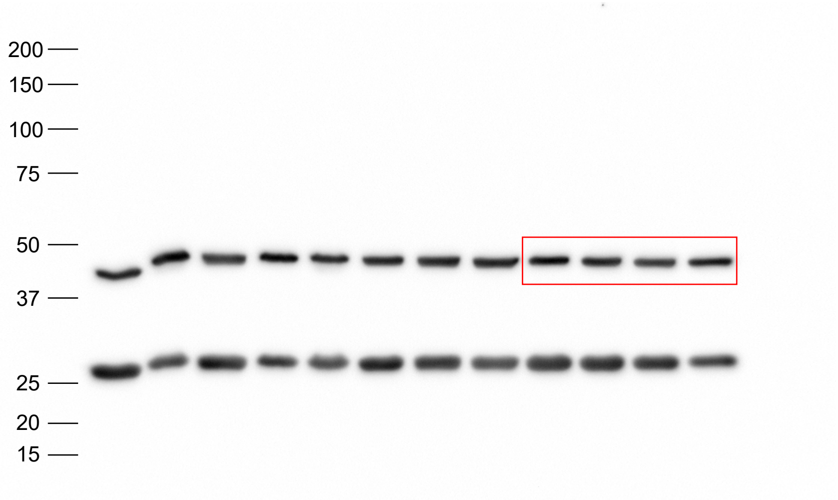

FLAG
(reprobed following Venus and β-Actin detection)

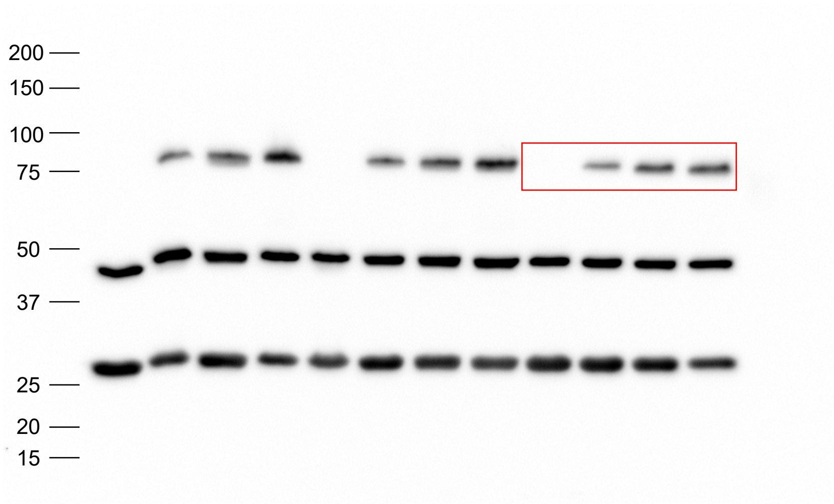

Source Data FS5

G Venus (Venus-3′UTR construct)

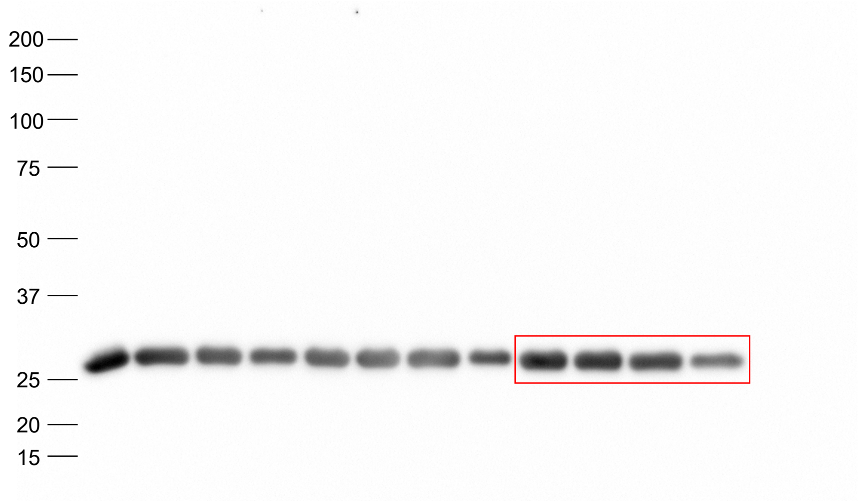

β-Actin
(reprobed following Venus detection)

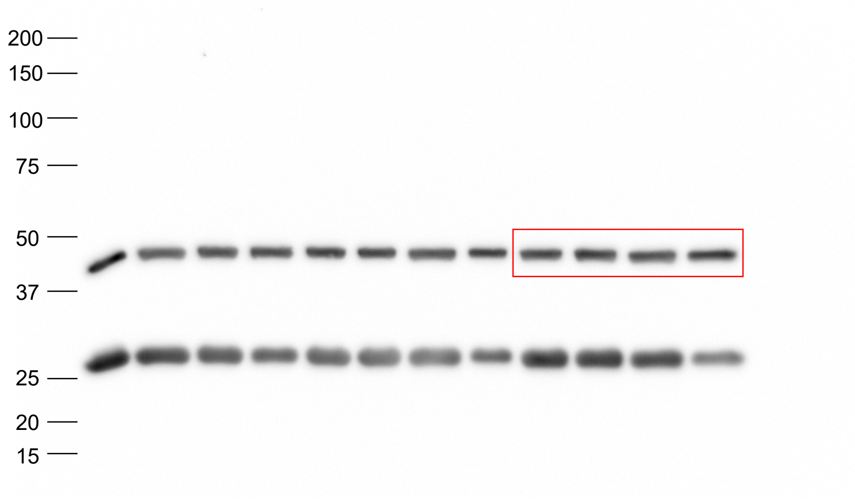

FLAG
(reprobed following Venus and β-Actin detection)

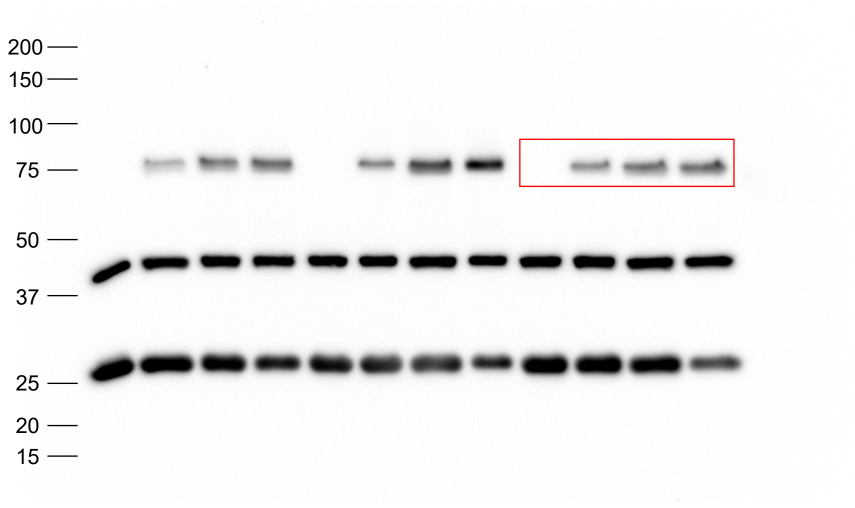

Supplement: SourceData FS5 — is the source file for Fig. S5. [file jcb_202406097_sourcedatafs5.pdf]
